# Cationic Sulfonium‐Based Tripodal Ligand and Its Rh(I) Complexes

**DOI:** 10.1002/chem.71052

**Published:** 2026-04-28

**Authors:** Nitsan Barel, Shrouq Mujahed, Kamal Uddin Ansari, Katarzyna Młodzikowska‐Pieńko, Renana Gershoni‐Poranne, Yuri Tulchinsky

**Affiliations:** ^1^ Institute of Chemistry, Edmond Safra Campus Hebrew University of Jerusalem Jerusalem Israel; ^2^ Schulich Faculty of Chemistry and the Resnick Sustainability Center for Catalysis Technion–the Israel Institute of Technology, Technion City Haifa Israel

**Keywords:** cationic ligands, Lewis‐acid catalysis, sulfonium cations, tripodal ligand frameworks

## Abstract

Herein we report the first cationic tripodal ligand—consisting of a sulfonium donor embedded in a tripodal scaffold—together with its *mono‐* and *bis‐*cationic Rh(I) complexes. To elucidate the distinctive properties of this new ligand, its Rh(I) carbonyl complex was subjected to comprehensive structural (XRD) and spectroscopic (NMR, IR) analyses, compared to the isostructural analogs with neutral P‐based and anionic Si‐based ligands. This comparative study revealed systematic trends along the series, including a progressive shortening of the E‐Rh bond (E = Si, P, S) and increasing blue shifts of the CO stretching frequencies. These observations were corroborated by DFT calculations, which also indicated a steady negative charge depletion on Rh along the series. Electrochemical studies (CV) further highlighted the effect of the cationic sulfonium donor, revealing an anodic shift of ∼1.4 V in the Rh(I) reduction potential, relative to the P‐based analog. Lewis acidity measurements of the coordinatively unsaturated Rh(I) complexes (by the Guttman‐Becket method) yielded acceptor numbers approaching those of polyfluoroaryl boranes for the sulfonium complex. Finally, by representative 2:1 condensations of N‐methylindole with carbonyl compounds, we demonstrated how the high Lewis acidity of the Rh(I) center, imparted by its sulfonium coordination, can be exploited in Lewis acid catalysis.

## Graphical abstract

We report a cationic EP_3_‐type tripodal ligand based on an aromatic sulfonium cation (E = S^+^) along with a series of its Rh(I) complexes. Structural, spectroscopic, electrochemical, and computational analyses of these complexes compared to isostructural analogs (E = Si^−^ or P) reveal pronounced electron density depletion at Rh in the sulfonium congener, which is effectively leveraged for Lewis acid catalysis.

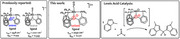

## 1 Introduction

**SCHEME 1:**
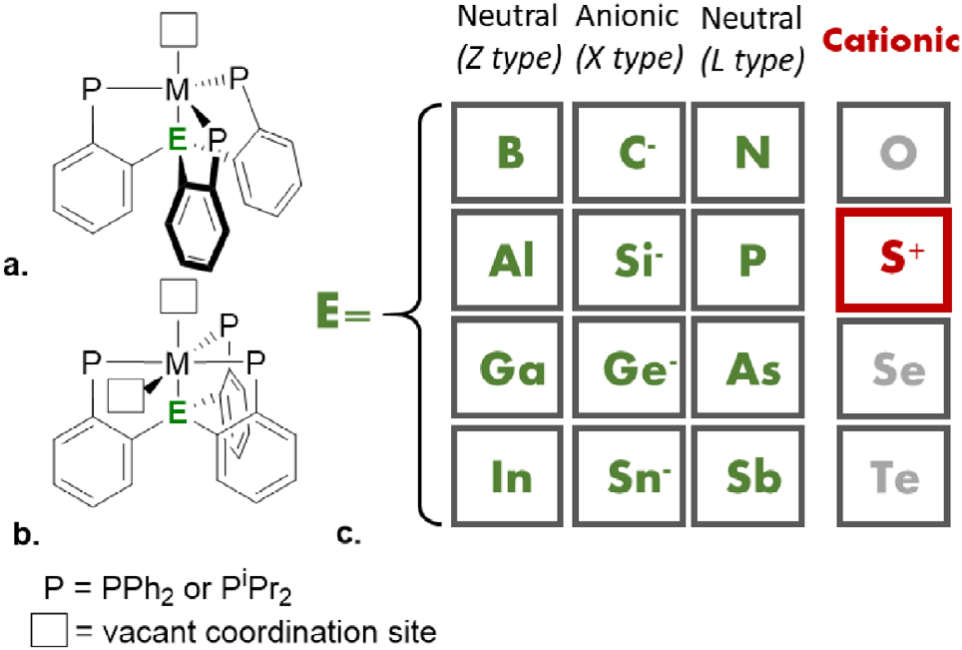
Trigonal pyramidal/bipyramidal (a) and octahedral (b) complexes of tripodal ligands and elements introduced at the apical position in these complexes (c).

Tripodal ligands, consisting of a central metal‐coordinating atom flanked by three chelating phosphine arms, are well‐known for their ability to create a rigid coordination environment around a metal center. Therefore, they have found extensive applications in homogeneous catalysis, including fundamental processes such as CO_2_ reduction [1] and N_2_ fixation [2]. These scaffolds are particularly effective at enforcing a trigonal pyramidal/bipyramidal (TP or TBP) coordination sphere (Scheme 1a), even on metal centers that typically adopt tetrahedral or square planar geometries (i.e., Cu(I), Pt(II), and so forth) [3, 4, 5]. Tripodal ligands can also adapt to octahedral metal centers by distorting from their inherent trigonal symmetry [6, 7, 8, 9]. In such cases, two *cis‐*oriented coordinated sites remain unoccupied, which is crucial for enabling the metal to engage in oxidative addition reactions [8] or *η* ^2^‐coordination of incoming substrates (Scheme 1b) [9].

Whereas the phosphine donors at the equatorial positions typically play a supporting role and do not have a strong effect on the metal, varying the apical atom does enable fine‐tuning of the electronic properties of the coordinated metal center. This is particularly important for catalysis, because the coordination site *trans* to the apical atom is the one most accessible to incoming substrates and therefore also the most strongly affected by its electronic character. In addition, the strong chelation by the equatorial donors can be used to enforce coordination of otherwise poorly coordinative species, facilitating detailed studies of their metal‐binding properties.

For these reasons, over the past decades, the family of isostructural tripodal ligands with this general skeleton has grown to include nearly all group 13–15 *p‐*block elements (except for the heaviest ones) as their apical atom, corresponding to the Z‐, X‐, and L‐type coordination modes according to the CBC classification (Scheme 1c) [10]. Within those, perhaps the most notable are the boron‐based tripodal ligands, first introduced and studied by Bourissou [3, 11, 12, 13]. Later on, Peters demonstrated that low‐valent Fe complexes of these ligands are among the very few artificial systems capable of catalyzing the reduction of N_2_ to ammonia [2]. Following this breakthrough, the Peters group also explored the catalytic performance of analogous tripodal ligands based on crystallogens (C, Si) [14, 15], and pnictogens (N) [16], and heavier icosagen congeners (Al, Ga) [17], all of which were found to be inferior to the original systems. In addition, the Si‐based ligands were successfully employed for catalytic hydrogenation of alkenes, alkynes, and CO_2_ [18, 19], as well as nitrene‐mediated homo‐coupling of aryl azides [20, 21].

Unlike the relatively recent tripodal ligands mentioned above, the corresponding phosphorus‐based analog—along with its heavier group 15 congeners (E = As, Sb)—has been present for decades, since first prepared by Venanzi in the 1960's [22, 23]. Yet, successful applications of the pnictogen‐based tripodal ligands in homogeneous catalysis have only emerged in recent years, when Beller [1, 24, 25, 26, 27] and others [28, 29] demonstrated the utility of tripodal tetraphosphines as supporting ligands for numerous important transformations, such as reduction and valorization of CO_2_ [1, 28], along with hydrogenation of α,β‐unsaturated aldehydes [24], nitro‐ and heteroarenes [25, 26, 27], and epoxides [29].

So far, all reported tripodal ligands were either neutral (groups 13 and 15) or negatively charged (group 14). However, extending this isostructural ligand series to the chalcogen elements (group 16) should give rise to positively charged tripodal ligands. Although still rare, cationic ligands have been attracting increasing interest beyond purely academic curiosity, due to the accumulating evidence of their efficacy in electrophilic catalysis [30, 31, 32, 33, 34, 35]. A key challenge in employing these ligands lies in their inherently poor coordinating ability, which compromises the stability of their metal complexes under catalytic conditions. This limitation is often overcome by incorporating the cationic donor into a chelating framework, for instance, a pincer‐type scaffold [36].

In recent years, our group has been actively involved in developing a new class of such pincer ligands, based on sulfonium cations [37, 38, 39, 40]. In the course of this work, we have established that aromatic sulfonium cations—previously not known to engage in metal coordination—can act as L‐type ligands [37]. Furthermore, we have shown that the positive charge on the sulfonium donor significantly improves the performance of the corresponding Pt(II) complexes as π‐acid catalysts, compared to that of a neutral thioether‐based ligand [39, 40]. Expanding the diversity of sulfonium ligand architectures beyond the tridentate pincer geometry represents a logical progression of our research. Herein, we report a tetradentate sulfonium ligand featuring a tripodal skeleton. In addition to extending the tripodal ligand series to the chalcogen group (S), it constitutes the first example of a cationic ligand with a tripodal geometry. Detailed spectroscopic, electrochemical, and computational studies on its Rh(I) complexes reveal that coordination to the sulfonium donor significantly enhances the electrophilicity of the metal center. We also show that this effect could be successfully leveraged for electrophilic (Lewis acid) catalysis.

## 2 Results and Discussion

### 2.1 Synthesis of the Tripodal Sulfonium Ligands

**SCHEME 2:**
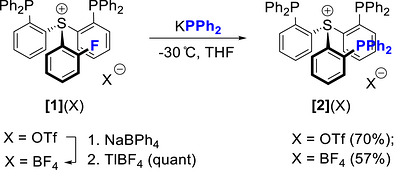
Synthesis of the tripodal sulfonium ligands **[2]**(OTf) and **[2]**(BF_4_). Yields are shown in brackets.

In our previous work [37], we reported sulfonium pincer ligand **[1]**(OTf), containing an *o‐*fluorophenyl motif (Scheme 2). While originally introduced as an NMR probe for monitoring sulfonium coordination, the fluorine substituent also offers synthetic utility, as it can serve as a leaving group. Indeed, aryl fluorides are well‐known to undergo nucleophilic aromatic substitution (S_N_Ar) with strong nucleophiles, especially when activated by an electron‐withdrawing substituent [41], such as the sulfonium group. We therefore reasoned that reacting **[1]**(OTf) with a diphenylphosphide nucleophile might afford a tripodal *tris‐*phosphine ligand. However, it was essential to ensure that the nucleophilic attack occurs at the *ortho‐*position, rather than at the *ipso*‐carbon, since the diphenylsulfide moiety can become a leaving group as well. After meticulous optimization of the reaction conditions (see Supporting Information), the desired tripodal ligand **[2]**(OTf) was obtained in a good yield (Scheme 2). An analogous ligand with a noncoordinative counter anion **[2]**(BF_4_) could be prepared by the same method, starting from the anion‐exchanged sulfonium pincer **[1]**(BF_4_).

**FIGURE 1:**
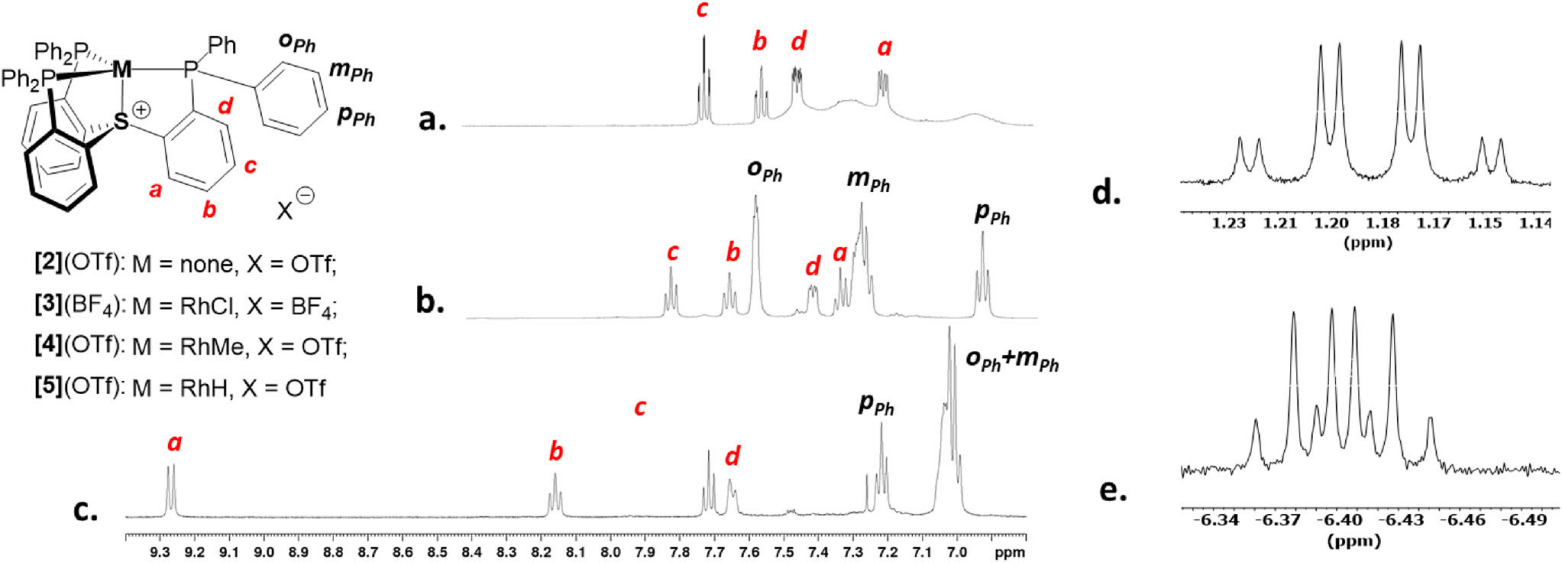
Low field region of the ^1^H NMR spectra of the tripodal sulfonium ligand **[2]**(OTf) at rt and −90°C (a and b, respectively) compared to its Rh(I) complex **[3]**(BF_4_) (c); and high field region of the ^1^H NMR spectra complexes **[4]**(OTf) (d), and **[5]**(OTf) (e), showing the methyl and hydride signals, respectively.

Ligand **[2]**(OTf) exhibits a sharp ^31^P{^1^H} NMR signal at δ = −14.3 ppm. In contrast, its ^1^H NMR spectrum displays several broad peaks at δ = 7.41–7.25 and 7.04–6.91 ppm, indicative of hindered rotation of the phenyl substituents about the C─P bonds (Figure 1a). Upon cooling to −90°C, this rotation is fully arrested, which resolves the phenyl proton resonances into 3 distinct signals at 7.61, 7.29, and 6.96 ppm, corresponding to the *ortho*, *meta*, and *para* positions, respectively (Figure 1b), as assigned by ^1^H‐^1^H COSY (Figure S39).

**FIGURE 2:**
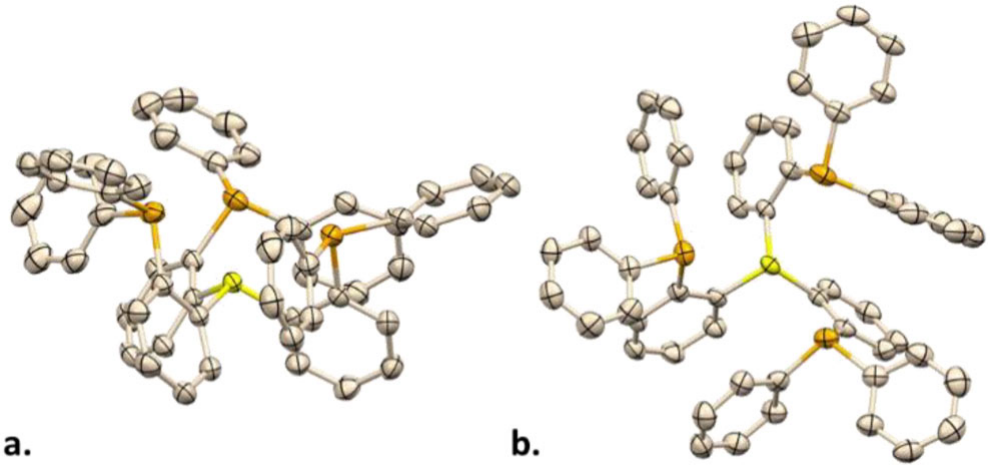
XRD structure of the tripodal sulfonium ligand **[2]**(OTf)–side view (a) and top view (b) are shown. The counterion, solvent molecules, and H atoms are omitted for clarity.

The XRD structure of **[2]**(OTf) revealed a *C*_3_‐symmetric propeller‐shaped molecule (Figure 2). This tripodal scaffold appears perfectly preorganized to stabilize a sulfonium‐metal bond, with all three phosphine donors facing the same side of the molecule with their lone pairs directed toward the same central point just above the sulfonium center.

### 2.2 Monocationic Rh(I) Complexes

To evaluate how the axial cationic donor of the new tripodal sulfonium ligands **[2]**(X) (X = OTf or BF_4_) affects their coordinative properties, we decided to focus on the Rh(I) complexes. This choice allows for a direct comparison not only to the known Rh(I) complexes of the structurally‐related tripodal ligands (*vide infra*), but also to our previous Rh(I) complexes with pincer‐sulfonium ligands.

**SCHEME 3:**
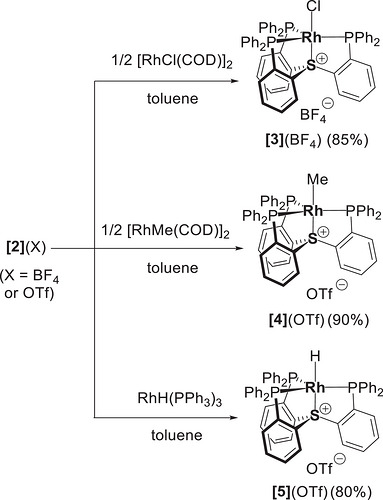
Synthesis of the monocationic Rh(I) complexes **[3]**(BF_4_), **[4]**(OTf), and **[5]**(OTf). Yields are shown in brackets.

To begin with, we aimed to synthesize a tripodal analog of the previously reported RhCl complex with ligand **[1]**(OTf) [37]. Addition of the [RhCl(COD)]_2_ precursor to a stirred suspension of ligand **[2]**(BF_4_) in toluene led to a gradual formation of dark purple solid, which was identified as the expected coordination product, **[3]**(BF_4_) (Scheme 3). ^31^P{^1^H} NMR spectrum of this compound confirmed the coordination of all three phosphines, as evidenced by a single doublet at δ = +41.5 ppm (^1^ *J*_P‐Rh_ = 130.5 Hz), consistent with a trigonal symmetry of this complex.

Metal coordination of sulfonium in this complex could be inferred from its ^1^H NMR, by the prominent downfield shifts of the S‐aryl protons at the *ortho* and *meta* positions, relative to the free ligand (Figure 1c). In our previous work with the pincer sulfonium ligand **[1]**(X), similar downfield shifts of the S‐aryl *ortho* protons served as a clear indication of a tridentate S‐bound—rather than a bidentate S‐free—coordination mode in the Pt(II) complexes [38].

**FIGURE 3:**
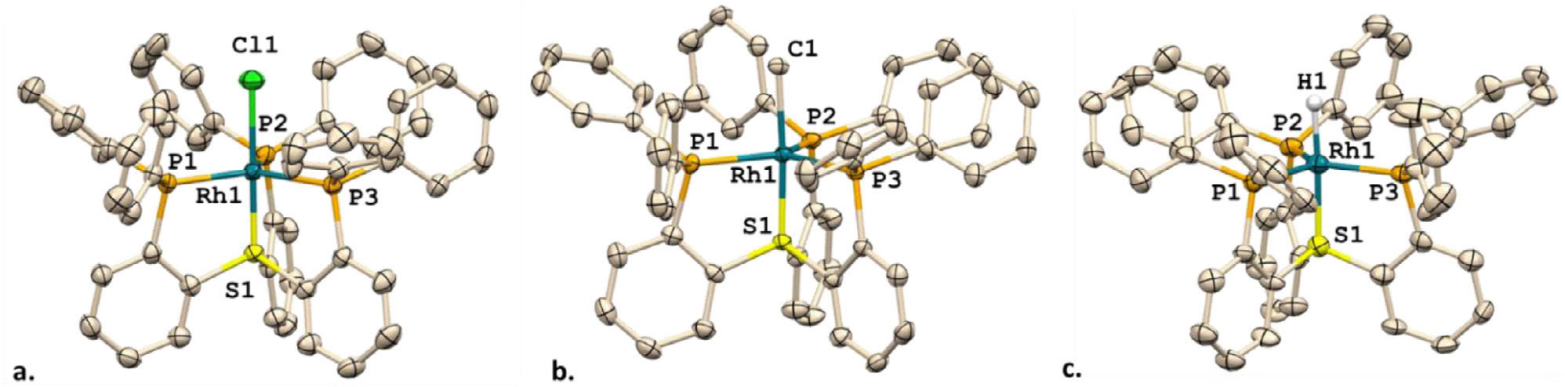
XRD structures of the mono‐cationic Rh(I) complexes **[3]**(BF_4_) (a), **[4]**(OTf) (b), and **[5]**(OTf) (c). Counterions, solvent molecules, and H atoms (except for the hydride) are omitted for clarity.

The structure of complex **[3]**(BF_4_) was subsequently confirmed by XRD, revealing the expected TBP geometry around the metal center (Figure 3a), with a short S‐Rh bond of 2.1144(9) Å. The S─C bonds of the coordinated sulfonium are slightly elongated compared to the free ligand (1.823 vs. 1.793 Å on av.), presumably due to the population of antibonding σ*_S‐C_ orbitals by π‐back donation with the Rh(I) center. Furthermore, to accommodate metal binding, the S‐aryl ‘blades’ rotate about the S─C bonds and align almost parallel to the S‐Rh axis, approaching an overall *C*_3v_ molecular symmetry.

Having established the coordination of sulfonium in **[3]**(BF_4_), which represented the first stable complex of a cationic tripodal ligand, we sought to prepare analogous Rh(I) complexes featuring a reactive functional group, such as an alkyl or a hydride, positioned *trans* to the sulfonium moiety, aiming at their potential application in electrophilic activation of C─H bonds. To this end, only a handful of Rh(I) hydride and Rh(I) alkyl complexes supported with a TBP geometry were reported [42, 43, 44]. Gratifyingly, such compounds, **[4]**(OTf) and **[5]**(OTf), could be easily prepared in good yields by treating ligand **[2]**(OTf) with the corresponding Rh(I) precursors, [Rh(Me)(COD)]_2_ or RhH(PPh_3_)_3_, respectively (Scheme 3). Similarly, to their RhCl congener, **[3]**(BF_4_), the ^31^P{^1^H} NMR spectra of both complexes displayed characteristic doublets at δ = 45.0 and 61.5 ppm, confirming their C_3_‐symmetric structure in solution. Notably, the ^31^P‐^130^Rh coupling constants were significantly larger (^1^ *J*_P‐Rh_ = 159.1 and 156.7 Hz, respectively), indicating higher electron density on the metal (*vide infra*), due to the stronger σ‐donor character of the methyl and hydride ligands, compared to chloride. The ^1^H NMR spectra of complexes **[4]**(OTf) and **[5]**(OTf) in the aromatic region were also quite similar to that of **[3]**(BF_4_), with strongly deshielded S‐aryl *ortho* protons, resonating at δ = 9.49 and 9.95 ppm, respectively (Figures S18 and S22). In addition, high field regions of the spectra displayed signals corresponding to Rh‐C ***H***_3_ (at δ = 1.19 ppm) and Rh‐***H*** (at δ = −6.36 ppm). Depending on the relative magnitudes of the coupling constants to ^103^Rh, these signals exhibited complex splitting patterns of doublets of quartets (dq, ^2^ *J*_H‐Rh_ = 2.2, ^3^ *J*_H‐P_ = 9.4 Hz, Figure 1d) or quartet of doublets (qd, ^1^ *J*_H‐Rh_ = 11.8, ^2^ *J*_H‐P_ = 7.4 Hz, Figure 1e). In addition, in case of **[4]**(OTf), the Rh‐***C*** H_3_ signal with a dq multiplicity (^1^ *J*_C‐Rh_ = 18.7, ^2^ *J*_C‐P_ = 7.2 Hz) was clearly visible in the ^13^C {^1^H} NMR spectrum at δ = −10.8 ppm (Figures S19 and S40).

Complexes **[4]**(OTf) and **[5]**(OTf) were further characterized by XRD (Figures 3b, 3c, respectively), enabling a detailed structural comparison with their RhCl analog (selected bond lengths and bond angles are given in Table S4, columns 2–4). Notably, the sulfonium‐Rh bonds in the former two complexes (2.1591(8) and 2.182(2) Å) are somewhat elongated compared to that of the latter (2.1144(9) Å), most likely because of the strong *trans*‐influence of the methyl and the hydride moieties. In contrast, the chloride ligand, being a π‐donor, engages in synergistic ‘push−pull’ interactions with the π‐acceptor sulfonium, which enhances the sulfonium‐metal π‐bonding and manifests in a very short S─Rh bond length. We have previously observed the same bond length pattern in complexes of pincer sulfonium ligands with PtMe and PtCl fragments [38].

### 2.3 *Bis*‐cationic Rh(I) Complexes

To further explore the coordinative behavior of this novel tripodal ligand, we aimed to test the stability of the sulfonium–metal bond upon converting the formally neutral Rh(I) fragment into a cationic one, either by occupying the axial coordination site with a neutral ligand or by leaving it vacant. The latter scenario is particularly relevant for a potential activation of small molecules through coordination to the metal center, which, due to its interaction with the sulfonium moiety, should become highly electrophilic.

**SCHEME 4:**
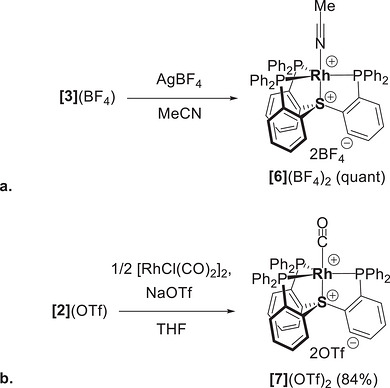
Synthesis of the *bis‐*cationic Rh(I) complexes **[6]**(BF_4_)_2_ (a) and **[7]**(OTf)_2_ (b) yields are shown in brackets.

We therefore carried out chloride abstraction from complex **[3]**(BF_4_) by treating it with AgBF_4_ in acetonitrile (Scheme 4a), which resulted in a quick color change of the reaction solution from dark purple to dark red. The ^31^P{^1^H} NMR spectrum of the reaction mixture indicated a quantitative conversion of the starting material into a single product, resonating at δ = +46.2 ppm (^1^ *J*_P‐Rh_ = 123.6 Hz). The ^1^H NMR spectrum of this new compound, **[6]**(BF_4_)_2_, isolated as a red crystalline powder, was also qualitatively similar to that of **[3]**(BF_4_), implying that the TBP geometry was retained during this transformation.

**FIGURE 4:**
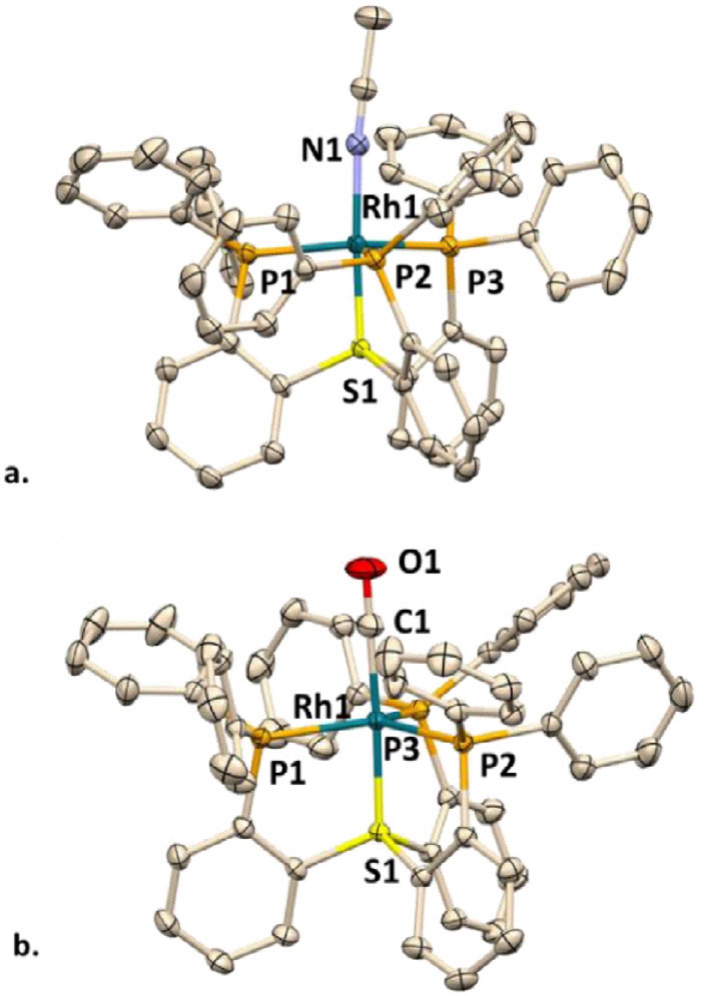
XRD structures of the *bis*‐cationic Rh(I) complexes **[6]**(BF_4_)_2_ (a) and **[7]**(OTf)_2_ (b). Counterions, solvent molecules, and H atoms are omitted for clarity.

The absence of a coordination shift or broadening of the BF_4_ anion signal in the ^19^F NMR ruled out its interaction with a coordinatively unsaturated Rh(I) center and therefore strongly suggested that the Rh(I) center in this complex is coordinatively saturated. This conjecture was confirmed by XRD, which revealed that the axial position *trans* to the sulfonium in **[6]**(BF_4_)_2_ is occupied by a solvent MeCN molecule (Figure 4a). The XRD also unequivocally established the presence of a cation‐cation bonding between the sulfonium moiety and the cationic Rh(I) fragment. Interestingly, the S─Rh bond length of 2.131(6) Å in this *bis‐*cationic complex (Table S4, entry 2) lies between those of the *mono‐*cationic RhCl and RhMe complexes. This finding is hardly surprising, as the MeCN ligand exerts a weaker *trans‐*influence compared to the methyl group in **[4]**(OTf) and also a weaker π‐donor compared to the chloride **[3]**(BF_4_).

Having thus confirmed cation‐cation bonding in **[6]**(BF_4_)_2_, we proceeded with the preparation of a *bis‐*cationic Rh(I)(CO) complex. Previously, Nakazawa used such Rh(I) carbonyl systems to study the donor/acceptor properties of isostructural tripodal *tris‐*phosphine ligands bearing boron [45] and group 14 elements (Si, Ge, Sn) [46] at the apical position, providing an excellent benchmark for a quantitative assessment of the electronic properties of sulfonium compared to neutral and anionic tripodal ligands.

Reacting ligand **[2]**(OTf) with a [RhCl(CO)_2_]_2_ precursor in the presence of NaOTf, as a chloride abstracting agent, resulted in a clean formation of the corresponding Rh(I) carbonyl complex, **[7]**(OTf)_2_ (Scheme 4b). The presence of a single coordinated CO molecule was evident from the IR spectrum exhibiting a sharp peak at 2030 cm^−1^ (Figure S59). This value is noticeably higher compared to most Rh(I) mono‐carbonyl complexes, even the positively charged ones [47, 48, 49, 50, 51], indicative of a highly electrophilic character of the Rh center in this complex *(vide infra)*.

Multinuclear NMR analysis of complex **[7]**(OTf)_2_ was consistent with the expected TBP coordination sphere, where a carbonyl group occupies the axial position *trans* to the sulfonium center (^31^P and ^13^C_CO_ chemical shifts and the corresponding coupling constants are given in Table 1). This structure was further confirmed by XRD (Figure 4b), which also revealed that the S─Rh bond in this complex is slightly elongated (2.2047(4) Å), presumably due to the competing interactions of two π‐acceptor ligands (sulfonium and CO) for electron density from the same metal orbital ($d_{z^{2}}$).

**TABLE 1:** Selected spectroscopic and structural data.

Entry	Parameter	10^a^	[8](OTf)	[7](OTf)_2_
1	ν_CO_(cm^−1^)	1958	2007	2030
2	δ ^13^C{^1^H} (ppm)	207.3	201.2	193.6
3	^1^ *J*_C‐Rh_(Hz)	nd	99.7	62.1
4	^1^ *J*_P‐Rh_(Hz)^b^	150.7	131.7	117.7
5	Rh‐E (Å)	2.3308(6)	2.3605(5)	2.2047(4)
6	Rh‐P (Å)^b^, ^c^	2.312(6)	2.344 (5)	2.360(4)
7	Rh‐C (Å)	1.921(3)	1.902(2)	1.8833(4)
8	C‐O (Å)	1.137(3)	1.122(3)	1.125(2)

^a^ Data taken from ref [46].

^b^ Equatorial phosphines.

^c^ Average value.

**SCHEME 5:**
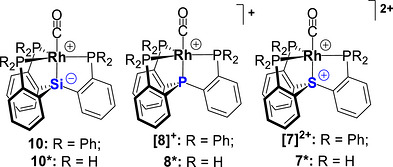
Isostructural series of Rh(I) carbonyl complexes with Si‐, P‐, and S‐based tripodal ligands (anions not shown). Model structures used in computational studies are designated with asterisks.

Complex **[7]**(OTf)_2_ extends the isoelectronic series of tripodal Rh(I) carbonyl complexes, following those of the anionic Si‐based ligand (studied by Nakazawa) [46], and of the neutral P‐based analog (Scheme 5). The latter complex was prepared by Venanzi et al. back in 1975 [52], but its full spectroscopic and structural characterization was unavailable. This prompted us to prepare complex **[8]**(OTf), following the same synthetic route as undertaken for **[7]**(OTf)_2_, starting from the commercially available *tris*‐(*o*‐(diphenylphosphino)‐phenyl)phosphine ligand, **9** (XRD structure is shown in Figure S76).

Full characterization of complexes **[7]**(OTf)_2_ and **[8]**(OTf), together with the available literature data for their Si‐based analog, **10** (Scheme 5), provides a rare opportunity to systematically study spectroscopic and structural trends arising from varying the apical donor atom in isoelectronic tripodal ligands across three successive third row elements (Si, P, and S), as summarized in Table 1.

In contrast to the small red shift of the CO stretching frequency observed by Nakazawa upon replacing the apical Si atom in **10** by its heavier congeners [46], changing the apical donor from Si^−^ to P and S^+^ results in a pronounced blue shift (Table 1, Entry 1). This trend clearly points out the decreasing π‐back donation from the metal to the π*_CO_ orbital, indicating that the Rh center becomes more electron‐deficient. Inspection of the carbonyl signals in the ^13^C{^1^H} NMR (Table 1, entry 2), however, might seem at odds with the IR data, as they shift upfield along the series, in contrast to the expected deshielding effect of the increasingly positive Rh center. However, the reduced electron density at carbon mostly affects the diamagnetic shielding term (*σ*_d_), while the screening tensor of metal carbonyls is dominated by the paramagnetic term (*σ*_p_), which has a negative sign. As a result, an increase in the CO bond order due to weaker back‐donation leads to a stronger paramagnetic shielding, manifesting in the upfield shift of the carbonyl resonance [53].

Further evidence for the progressive electron depletion at Rh comes from the ^1^ *J*_C‐Rh_ coupling constants, which decrease along the series (Table 1, entry 3). The same trend becomes even more obvious when comparing the values of ^1^ *J*_P‐Rh_ constants, which also decline consistently (Table 1, entry 4). A related phenomenon is well documented in Pt chemistry, where isostructural Pt‐phosphine complexes show a clear correlation between the electron density on the metal and ^1^ *J*_P‐Pt_ coupling constant values [54, 55, 56]. With the smallest ^1^ *J*_P‐Rh_ of only 117.7 Hz, **[7]**(OTf)_2_ stands out as the most electron‐deficient Rh complex, not only relative to its Si and P analogs, but also compared to other tripodal sulfonium complexes reported herein.

Structural comparison reveals a noticeable shortening of the Rh─E bond along the series (E = Si, P, S), probably due to the enhanced Rh‐to‐E π‐back donation, although it could also stem from the contraction of covalent radius of the E atom across the period (Table 1, entry 5). This shortening of the E‐Rh bond is accompanied by a slight elongation of the three Rh─P_eq_ bonds (on average), likely in order to relieve the structural strain caused by the tighter binding of the metal to the apical atom (Table 1, entry 6). Surprisingly, contrary to the expected inverse correlation between the carbonyl bond order and the Rh‐C_CO_ bond length (as suggested by the canonical M─C≡O and M═C═O resonance structures), the Rh─C_CO_ bond actually contracts along the series (Table 1, entry 7). Most likely, this is a consequence of the progressively weaker *trans‐*influence of the apical donor.

To investigate the E‐Rh bonding and charge distribution within this series of complexes, we turned to DFT calculations on model complexes 7*, 8*, and 10*, where the phenyl substituents on the equatorial phosphines were replaced with H atoms (for the computational details, see Supporting Information). The optimized geometries showed good agreement with the XRD data, reproducing the observed structural trends—namely, the progressive shortening of both E─Rh and C≡O bonds along the series (Table S7). Surprisingly, the calculated Wiberg bond indices for the E─Rh interactions did not follow the corresponding bond lengths, with the shortest S─Rh bond exhibiting the smallest bond order, consistent with sulfonium being the weakest donor in the series (Table S10, columns 2–4).

Another counterintuitive trend emerged from the Natural Population Analysis [57] (NPA): despite the formal charge on the E atoms varying from −1 to +1, in all three complexes, these donor atoms were found to be positively charged (Table S9, columns 2–4). This result fully aligns with previous calculations by Nakazawa [45], which showed that the Si─Rh bond in complex **10** is strongly polarized toward Rh. Similar polarization of the Si─Rh bond was also found in our model complex 10*, indicating that coordination of the formally anionic Si donor to the cationic Rh center results in a significant charge redistribution. Similar charge migration toward Rh occurs also in the P and S congeners, 8* and 7*, respectively, but to a lesser extent.

Consistently, the Natural Localized Molecular Orbital [58] (NLMO) calculations revealed polarity inversion of the E‐Rh bond, shifting along the series from Rh‐polarized to E‐polarized (Table S11). Most likely, this phenomenon arises from the increasing electronegativity of the E atoms, ranging from 1.90 for Si to 2.58 for S, relative to that of Rh (2.28). This also might explain why, despite the stepwise increase of the overall positive charge of the complexes, the local positive charge on the E atom decreases along the series.

After establishing the above spectroscopic and structural trends, we focused on assessing the impact of the cationic sulfonium center on the redox potential and Lewis acidity of the coordinated Rh(I) center, with the neutral phosphine‐based analog serving as a reference.

**FIGURE 5:**
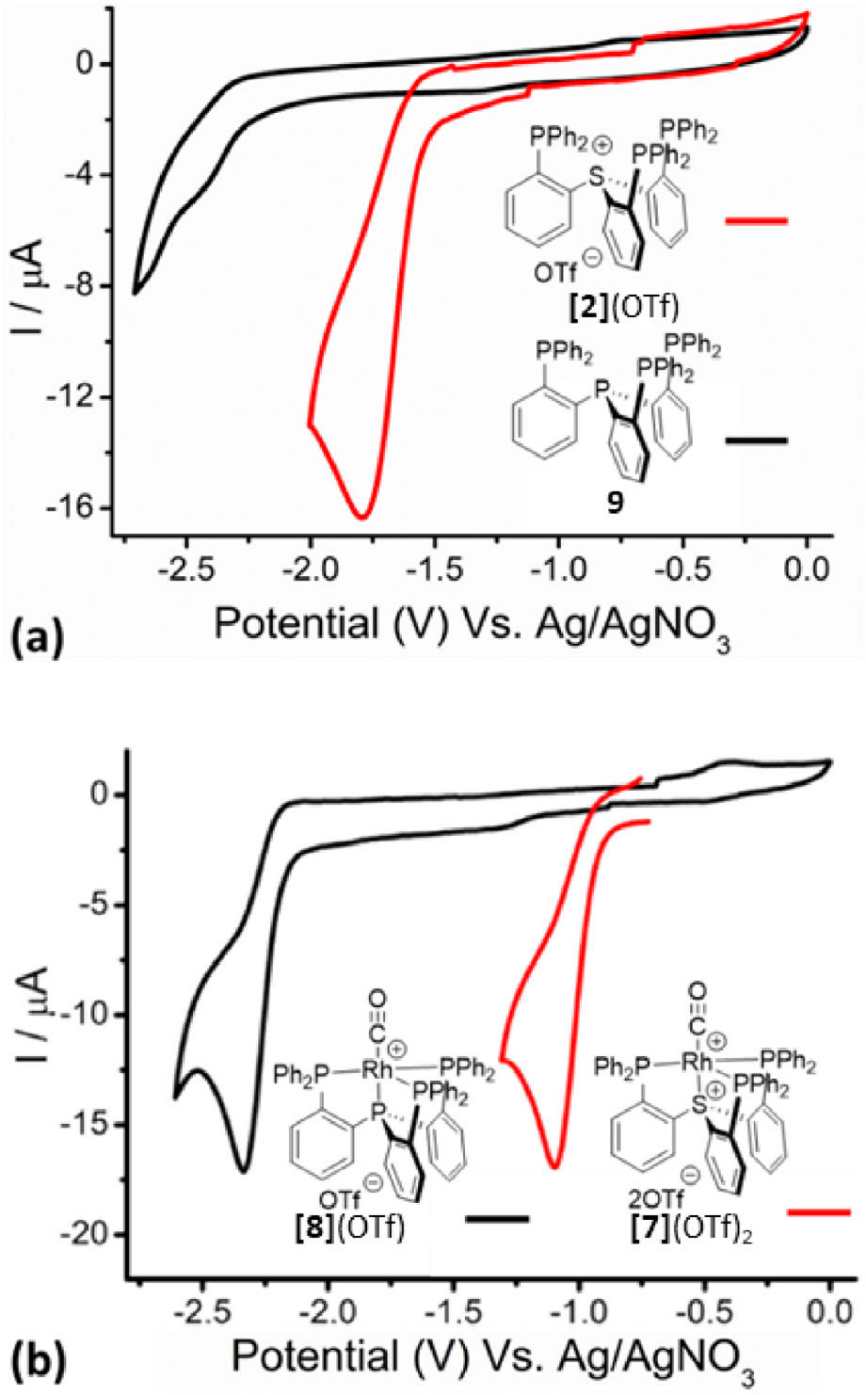
Cyclic voltammograms of the tripodal sulfonium‐ and phosphine‐based ligands **[2]**(OTf) and **9**, (a) and their Rh(CO) complexes, **[7]**(OTf)_2_ and **[8]**(OTf), (b) in THF. Conditions: (0.25 mM) under N_2_ atmosphere in THF at scan rate of 0.1 V/s.

First, we recorded cyclic voltammetry (CV) of the free ligands, **[2]**(OTf) and **9**, in THF. The reduction potential of **9** lies close to or even beyond the cathodic limit of THF, and therefore its cathodic wave is obscured by the reductive decomposition of the solvent (Figure 5a, black trace). Ligand **[2]**(OTf), on the other hand, exhibits a well‐defined cathodic peak at *E*_c_ = −1.78 V (Figure 5a, red trace), close to the parent triphenylsulfonium cation (*E*_c_ = −1.5 V) [59]. This reduction wave is irreversible, presumably due to the formation of a short‐lived neutral sulfonyl radical species, which decomposes rapidly into the corresponding aryl radical and diarylsulfide [60].

Next, we explored the reduction of the corresponding Rh(CO) complexes, **[8]**(OTf) and **[7]**(OTf)_2_ under the same conditions (Figure 5b, black and red traces, respectively). In this case, both compounds exhibited irreversible reduction waves, most likely corresponding to the Rh(I)/Rh(0) redox couple [61]. Notably, the reduction wave of the sulfonium complex occurs at a significantly less negative potential than that of its phosphine analog (*E*_c_ = −1.01 V vs. −2.44 V), demonstrating how coordination of the metal to the cationic sulfonium group facilitates its reduction (Figure 5b, red trace). Following these electrochemical studies, we attempted to carry out a chemical reduction of **[7]**(OTf)_2_ with Na/Hg or KC_8_. Unfortunately, all our attempts to trap the putative Rh(0) species at low temperature and to characterize it by EPR were unsuccessful.

To quantitatively assess the Lewis acidity of sulfonium‐coordinated Rh(I) center, we employed the Gutmann–Beckett method (GB), which relies on the complexation of phosphine oxides [62, 63]. In addition to triethylphosphine oxide (TEPO), the ^31^P{^1^H} NMR probe traditionally used in this method, we also employed a more sterically hindered triphenylphoshine oxide (TPPO), which has also been employed in the literature for the Lewis acidity measurements [64], despite being less sensitive to perturbations upon cation binding, compared to the former [65].

**SCHEME 6:**
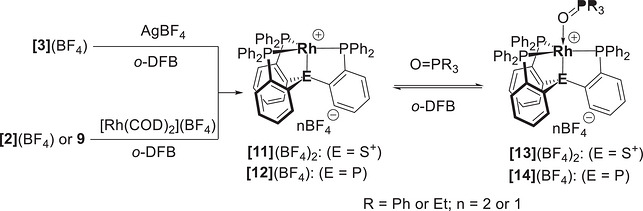
Preparation of coordinatively unsaturated Rh(I) complexes of the tripodal sulfonium‐ and phosphine‐based ligands and their complexation with phosphine oxides.

**FIGURE 6:**
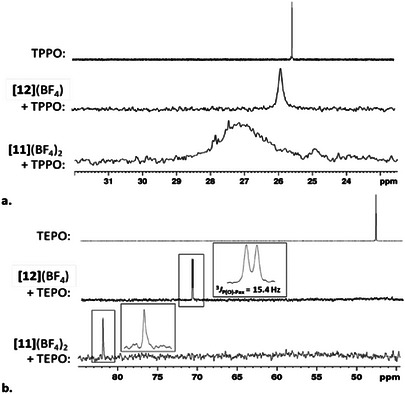
Comparing Lewis‐acidity of the cationic Rh(I) center in complexes **[11]**(BF_4_)_2_ and **[12]**(BF_4_) generated in situ by the GB method with TPPO (a) and TEPO (b).

The coordinatively unsaturated Rh(I) species **[11]**(BF_4_)_2_ for these studies was initially formed by chloride abstraction from complex **[3]**(BF_4_) (Figures S41 and S42). Later on, we found that the same species can also be prepared directly from the sulfonium ligand **[2]**(BF_4_) and a cationic Rh(I) precursor, [Rh(COD)_2_](BF_4_). The same strategy was also used for the preparation of the corresponding phosphine analogue, **[12]**(BF_4_), from ligand **9** (Scheme 6). Due to their limited stability, complexes **[11]**(BF_4_)_2_ and **[12]**(BF_4_) were not isolated and generated in situ prior to the GB measurements. Moreover, to avoid any undesired reactivity of their coordinatively unsaturated Rh(I) center, *o‐*difluorobenzene (*o*‐DFB), a noncoordinative and nonchlorinated solvent, was used for these studies. As evident from Figure 6a, interaction with the coordinatively unsaturated Rh(I) centers of the phosphine‐ and sulfonium‐based complexes **[12]**(BF_4_) and **[11]**(BF_4_)_2_ results in relatively minor upfield shifts of the TPPO signal (∆δ = 0.35 and 1.63 ppm, respectively), but in a significant peak broadening—especially for the sulfonium complex—indicative of a reversible complexation with a slow exchange between the free and coordinated TPPO (Table 2, entries 1–3). While the difference in coordination shifts between the two complexes is small, it is not negligible. In fact, a comparable difference in ∆δ values (∆δ = 0.12 vs. 3.23 ppm) was previously observed by Toste [66] for the coordinatively unsaturated cationic Au(I) and Au(III) complexes (respectively), despite the markedly higher oxophilicity of the Au(III) congener.

**TABLE 2:** Lewis acidity measurements of complexes **[12]**(BF_4_) and **[11]**(BF_4_)_2_ in *o‐*DFB by the Gutmann–Beckett method.^a^

Entry	Compounds	δ ^31^P{^1^H} (ppm)	W_1/2_ (Hz)	∆δ ^31^P{^1^H} (ppm)	AN
1	TPPO	25.52	1.0	—	—
2	TPPO + **[12]**(BF_4_)	25.87	24	0.35	—
3	TPPO + **[11]**(BF_4_)_2_	27.15	376	1.63	—
4	TEPO	47.82	5.3	—	13.6^b^
5	TEPO + **[12]**(BF_4_)	71.02	23.4	23.20	51.3
6	TEPO + **[11]**(BF_4_)_2_	81.88	7.6	34.06	75.3

^a^ See Section S2.3 for reaction procedures.

^b^ This value represents the acceptor number of the *o‐*DFB solvent.

In contrast, with TEPO, sharp signals exhibiting significantly larger coordination shifts were observed, suggested formation of well‐defined TEPO adducts, **[13]**(BF_4_)_2_ and **[14]**(BF_4_) (Figure 6b and Table 2, entries 4–6). Additional structural information about these adducts could be inferred from the shape of the TEPO signal of the phosphine‐based adduct **[14]**(BF_4_). It appears as a doublet, due to magnetic coupling with the axial P atom (^3^ *J*_P‐P_ = 15.4 Hz), suggesting that in both adducts TEPO coordinates *trans* to the apical donor.

Acceptor numbers (AN), introduced by Gutmann [67], provide an excellent quantitative measure of Lewis acidity. On this scale, the phosphine complex is comparable to relatively mild Lewis acids, such as BiCl_3_ [68], SnCl_4_ [69] or mono‐cationic Mg(II) complexes [70]. In contrary, the AN of the sulfonium complex falls within the typical range of (polyfluoroaryl)boranes (70–82) [71], which are well‐known for their high Lewis acidity. This further demonstrates how coordination of a coordinatively unsaturated Rh(I) center to a sulfonium ligand significantly enhances its Lewis‐acidic character.

## 3 Application of the Tripodal Sulfonium Ligand in Catalysis

**SCHEME 7:**
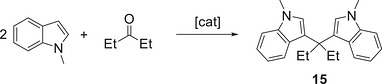
Representative Lewis‐acid catalyzed condensation reaction used for catalytic activity evaluation.

Encouraged by the high AN value determined for the sulfonium complex **[** **11** **]**(BF_4_)_2_, we wondered whether the enhanced Lewis acidity of its Rh(I) center can be leveraged for catalysis. To explore this possibility, we chose a representative reaction, namely, a 2+1 indole‐ketone condensation, a well‐established Lewis acid‐catalyzed transformation [72]. For the initial evaluation, N‐methylindole and 3‐pentanone were chosen as model substrates (Scheme 7). Taking advantage of 3‐pentanone being a liquid, these reactions were carried out under neat conditions, with the ketone serving both as a solvent and as a reactant.

In the presence of 2 mol% of **[2]**(BF_4_) and [Rh(COD)_2_](BF_4_), we observed a 43% conversion of the starting indole into the doubly‐condensed product **15** after heating to 50 °C for 24 h. Full conversion was achieved by increasing the catalyst loading to 3 mol% (Table 3, entries 1 and 2). Alternatively, employing complex **[3]**(BF_4_) in the presence of a chloride abstraction agent (AgBF_4_) was equally effective, strongly supporting that the catalytically active species is indeed the *bis*‐cationic sulfonium complex **[11]**(BF_4_)_2_ formed in situ (entry 3). Control experiments confirmed that no product forms under identical conditions in the absence of catalyst. Likewise, no reaction was observed with **[2]**(BF_4_) alone, whereas the cationic Rh(I) precursor without the sulfonium ligand afforded only a negligible conversion (Entries 4–6).

**TABLE 3:** Evaluating the efficiency of sulfonium ligand in a representative Rh(I)‐catalyzed double‐condensation reaction.

Entry	Catalyst^a^	Loading(mol %)	Conversion(%)^b^
1	**[2]**(BF_4_) + [Rh(COD)_2_](BF_4_)	2	43%
2	**[2]**(BF_4_) + [Rh(COD)_2_](BF_4_)	3	100%
3	**[3]**(BF_4_) + AgBF_4_	3	100%
4	None	—	No reaction
5	**[2]**(BF_4_) only	3	No reaction
6	[Rh(COD)_2_](BF_4_) only	3	2%
7	**9** + [Rh(COD)_2_](BF_4_)	3	No reaction
8	HBF_4_*OEt_2_	3	100%^c^

^a^ See Section S2.4 for reaction conditions.

^b^ Determined by ^1^H NMR.

^c^ No formation of **15** was observed; instead, compound **16** was obtained as the major product.

To confirm the crucial role of the sulfonium moiety for the observed catalysis, we replaced the sulfonium ligand **[2]**(BF_4_) with its phosphine‐based counterpart **9**, which resulted in no catalytic reaction (entry 7). Finally, to rule out that the observed catalytic activity arises from an acid generated via thermal or photochemical decomposition of the sulfonium ligand [73] or its complex, we conducted a control experiment by replacing our Lewis acid catalyst with a strong Brønsted acid (HBF_4_*OEt_2_, entry 8). Although full conversion of the starting indole was observed, no formation of product **15** took place in this case. Instead, a mixture of several compounds was obtained, from which a major product with a molecular mass of 267.20 au (by HRMS) could be isolated in 42% yield. The exact structure of this compound was then established by multinuclear 2D NMR spectroscopy and identified as the 1+2 double condensation product **16** (for full structural assignment see Tables S1 and S2).


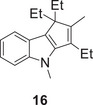


**SCHEME 8:**
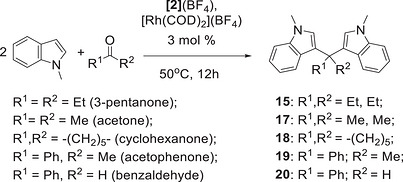
Representative Lewis‐acid catalyzed condensation reactions tested.

To evaluate the scope of our catalytic system, we examined several additional substrates (Scheme 8). With acetone, an excellent isolated yield, identical to that with the 3‐pentanone, was obtained (Table 4, entries 1 and 2). Remarkably, reactions with the more sterically demanding ketones, i.e., cyclohexanone and acetophenone, afforded a nearly quantitative yield of the corresponding condensation products **18** and **19**, respectively (entries 3 and 4). Upon cooling the reaction solution to room temperature after completion of the reaction, the acetophenone‐derived product (**19**) spontaneously formed colorless cubic crystals, allowing us to confirm its structure by XRD (Figure S77). Finally, benzaldehyde similarly could be used as a substrate, resulting in the corresponding product **20** in an excellent yield (entry 5).

**TABLE 4:** Reactivity scope of the tripodal sulfonium‐based Rh(I) catalyst.

Entry	Substrate^a^	Product	Conversion/Isolated yield(%)
1	3‐Pentanone	**15**	100/93
2	Acetone	**17**	100/93
3	Cyclohexanone	**18**	100/98
4	Acetophenone	**19**	100/96
5	Benzaldehyde	**20**	100/94

^a^ See Section S2.4 for reaction conditions.

While our primary goal in exploring these benchmark reactions was to provide a proof‐of‐concept demonstration that sulfonium‐induced Lewis acidity at the metal center can be leveraged for catalysis, it is noteworthy that compared to several reported protocols employing simple Lewis‐acidic metal salts, such as ZrCl_4_ [74] and Bi(NO_3_)_3_*5H_2_O [75], or nonmetal Lewis acids, such as B(C_6_F_5_)_3_ and PhSiCl_3_ [76], our catalytic system operates at a lower catalyst loading (3 mol% vs. 10 mol%) and, in one case, also at a lower temperature (50°C vs. 80°C) [76].

To support the experimental findings, we also investigated computationally how the tripodal ligand affects the activation of the Rh‐coordinated ketone substrate. Specifically, we optimized the geometry of model complexes 21*, 22*, and 23* (Figure S78), showcasing the interaction of a ketone molecule (acetone) with a coordinatively unsaturated Rh(I) center complexed by Si‐, P‐ and S‐based tripodal ligands. As observed in their carbonyl analogs, the E─Rh bond becomes progressively shorter across the series (Table S8), accompanied by a bond‐polarization shift from Rh‐centered to E‐centered character (Table S12). An additional structural trend present along the series is a marked contraction of the Rh─O_Acetone_ bond concomitant with a noticeable elongation of the C═O_Acetone_ bond (Table S8). Likewise, despite only a negligible increase in the positive charge on the carbonyl carbon (Table S9, columns 5–7), Wiberg bond indices indicate a significant weakening of the carbonyl bond order (Table S10, columns 5–7), consistent with an enhanced activation toward a nucleophilic attack, being most pronounced for the S‐based tripodal ligand.

## 4 Summary

In summary, in this work, we presented the first example of a positively charged tripodal ligand framework featuring a chalcogen element (sulfur) at its apical position. It's influence on the electronic properties and catalytic activity of a coordinated Rh(I) cation through combined experimental and computational studies. Spectroscopic, electrochemical, and computational data indicated that the sulfonium ligand significantly enhances the electrophilicity of the Rh(I) center, compared to isoelectronic silicon‐ and phosphorus‐based analogs, thereby enabling its successful application in electrophilic catalysis. Further exploration of this novel ligand platform for other types of catalytic reactions is currently underway in our lab.

## Conflicts of Interest

The authors declare no conflicts of interest.

## Supporting information




**Supporting File**: Additional supporting information can be found online in the Supporting Information section. The authors have cited additional references within the Supporting Information [74, 75, 76, 77, 78, 79, 80, 81, 82, 83, 84, 85, 86].
