# Supplementary material for: Cationic Sulfonium‐Based Tripodal Ligand and Its Rh(I) Complexes
Source: Chemistry. 2026 Apr 28;32(30):e71052. doi: 10.1002/chem.71052 (PMC13472339; doi:10.1002/chem.71052)
Supplement: Supplementary file 1 [file CHEM-32-e71052-s001.docx]

**Supporting Information**

Cationic Sulfonium-based Tripodal Ligand and its Rh(I) Complexes

Nitsan Barel,^[a]^ Shrouq Mujahed,^[a]^ Kamal Uddin Ansari,^[a]^ Katarzyna Młodzikowska-Pieńko,^[b]^ Renana Gershoni-Poranne,*^[b]^ and Yuri Tulchinsky*^[a]^

[a]	Mr. Nitsan Barel, Dr. Shrouq Mujahed, Dr. Kamal Uddin Ansari and Dr. Yuri Tulchinsky
Institute of Chemistry, Hebrew University of Jerusalem, Jerusalem 9190401, Israel
E-mail: [yuri.tulchinsky@mail.huji.ac.il](mailto:yuri.tulchinsky@mail.huji.ac.il)

[b]	Dr. Katarzyna Młodzikowska-Pieńko and Dr. Renana Gershoni-Poranne
Schulich Faculty of Chemistry, Technion – the Israel Institute of Technology, Haifa 3200003, Israel
E-mail: [rporanne@technion.ac.il](mailto:rporanne@technion.ac.il)

**Table of Contents**

[1.	General information	6](#_Toc225599206)

[2.	Synthetic procedures	7](#_Toc225599207)

[2.1	Preparation of tripodal sulfonium ligands	7](#_Toc225599208)

[2.2	Preparation of Rh(I) complexes of tripodal sulfonium and phosphine ligands	8](#_Toc225599209)

[2.3	Gutmann-Beckett method measurements of cationic Rh(I) sulfonium and phosphine complexes	12](#_Toc225599210)

[2.4	Optimized procedure for the catalytic reaction	12](#_Toc225599211)

[3.	NMR spectra	14](#_Toc225599212)

[Figure S1. ^1^H NMR (500 MHz) of [1](BF_4_) in CD_3_CN	14](#_Toc225599213)

[Figure S2. ^13^C{^1^H) NMR (125 MHz) of [1](BF_4_) in CD_3_CN	15](#_Toc225599214)

[Figure S3. ^31^P{H} NMR (162 MHz) of [1](BF_4_) in CDCl_3_	16](#_Toc225599215)

[Figure S4. ^19^F NMR (471 MHz) of [1](BF_4_) in CDCl_3_	16](#_Toc225599216)

[Figure S5. ^1^H NMR (500 MHz) of [2](OTf) in acetone-d_6_	17](#_Toc225599217)

[Figure S6. ^13^C{^1^H) NMR (125 MHz) of [2](OTf) in acetone-d_6_	18](#_Toc225599218)

[Figure S7. ^31^P{H} NMR (202 MHz) of [2](OTf) in acetone-d_6_	19](#_Toc225599219)

[Figure S8. ^19^F NMR (471 MHz) of [2](OTf) in acetone-d_6_	19](#_Toc225599220)

[Figure S9. ^1^H NMR (500 MHz) of [2](OTf) in acetone-d_6_ at -90.0±1.00°C	20](#_Toc225599221)

[Figure S10. ^1^H NMR (500 MHz) of [2](BF_4_) in acetone-d_6_	21](#_Toc225599222)

[Figure S11. ^13^C{^1^H) NMR (125 MHz) of [2](BF_4_) in acetone-d_6_	22](#_Toc225599223)

[Figure S12. ^31^P{H} NMR (162 MHz) of [2](BF_4_) in acetone-d_6_	23](#_Toc225599224)

[Figure S13. ^19^F NMR (376 MHz) of [2](BF_4_) in acetone-d_6_	23](#_Toc225599225)

[Figure S14. ^1^H NMR (500 MHz) of [3](BF_4_) in CDCl_3_	24](#_Toc225599226)

[Figure S15. ^1^*^3^*C{^1^H) NMR (*125* MHz) of [3](BF_4_) in CDCl_3_	25](#_Toc225599227)

[Figure S16. ^31^P{H} NMR (162 MHz) of [3](BF_4_) in CDCl_3_	26](#_Toc225599228)

[Figure S17. ^19^F NMR (376 MHz) of [3](BF_4_) in CDCl_3_	26](#_Toc225599229)

[Figure S18. ^1^H NMR (500 MHz) of [4](OTf) in CDCl_3_ at -30.0±1.00 °C	27](#_Toc225599230)

[Figure S19. ^1^*^3^*C{^1^H) NMR (*125* MHz) of [4](OTf) in CDCl_3_ at -30.0±1.00 °C	28](#_Toc225599231)

[Figure S20. ^31^P{H} NMR (162 MHz) of [4](OTf) in CDCl_3_	29](#_Toc225599232)

[Figure S21. ^19^F NMR (376 MHz) of [4](OTf) in CDCl_3_	29](#_Toc225599233)

[Figure S22. ^1^H NMR (500 MHz) of [5](OTf) in THF-d_8_	30](#_Toc225599234)

[Figure S23. ^13^C{^1^H) NMR (125 MHz) of [5](OTf) in THF-d_8_	31](#_Toc225599235)

[Figure S24. ^31^P{H} NMR (162 MHz) of [5](OTf) in THF-d_8_	32](#_Toc225599236)

[Figure S25. ^19^F NMR (376 MHz) of [5](OTf) in THF-d_8_	32](#_Toc225599237)

[Figure S26. ^1^H NMR (500 MHz) of [6](BF_4_)_2_ in CD_3_CN	33](#_Toc225599238)

[Figure S27. ^1^*^3^*C{^1^H) NMR (125 MHz) of [6](BF_4_)_2_ in CD_3_CN	34](#_Toc225599239)

[Figure S28. ^31^P{H} NMR (202 MHz) of [6](BF_4_)_2_ in CD_3_CN	35](#_Toc225599240)

[Figure S29. ^19^F NMR (470 MHz) of [6](BF_4_)_2_ in CD_3_CN	35](#_Toc225599241)

[Figure S30. ^1^H NMR (500 MHz) of [7](OTf)_2_ in CDCl_3_	36](#_Toc225599242)

[Figure S31. ^13^C{^1^H) NMR (125 MHz) of [7](OTf)_2_ in CDCl_3_	37](#_Toc225599243)

[Figure S32. ^31^P{H} NMR (202 MHz) of [7](OTf)_2_ in CD_2_Cl_2_	38](#_Toc225599244)

[Figure S33. ^19^F NMR (470 MHz) of [7](OTf)_2_ in CD_2_Cl_2_	38](#_Toc225599245)

[Figure S34. ^1^H NMR (500 MHz) of [8](OTf) in CD_2_Cl_2_	39](#_Toc225599246)

[Figure S35. ^1^*^3^*C{^1^H) NMR (*125* MHz) of [8](OTf) in CD_2_Cl_2_	40](#_Toc225599247)

[Figure S36. ^31^P{H} NMR (162 MHz) of [8](OTf) in CD_2_Cl_2_	41](#_Toc225599248)

[Figure S37. ^19^F NMR (376 MHz) of [8](OTf) in CD_2_Cl_2_	41](#_Toc225599249)

[Figure S38. Varianable temperature ^1^H NMR (500 MHz) of [2](OTf) in acetone-d_6_	42](#_Toc225599250)

[Figure S39. ^1^H-^1^H COSY NMR (500 MHz) [2](OTf) in acetone-d_6_ at -40.0±1.00°C	43](#_Toc225599251)

[Figure S40. ^1^H-^13^C HSQC NMR (500 MHz) of [4](OTf) in CDCl_3_ at -30.0±1.00°C	44](#_Toc225599252)

[Figure S41. ^31^P{H} NMR (202 MHz) of [11](BF_4_)_2_ from a reaction between [3](BF_4_) and AgBF_4_ in C_6_D_4_Cl_2_.	45](#_Toc225599253)

[Figure S42. ^1^H NMR (500 MHz) of [11](BF_4_)_2_ from a reaction between [3](BF_4_) and AgBF_4_ in C_6_D_4_Cl_2_	46](#_Toc225599254)

[Figure S43. ^1^H NMR (400 MHz) of 15 in CDCl_3_	47](#_Toc225599255)

[Figure S44. ^13^C{^1^H) NMR (100 MHz) of 15 in CDCl_3_	47](#_Toc225599256)

[Figure S45. ^1^H NMR (500 MHz) of 17 in CDCl_3_	48](#_Toc225599257)

[Figure S46. ^13^C{^1^H) NMR (125 MHz) of 17 in CDCl_3_	48](#_Toc225599258)

[Figure S47. ^1^H NMR (500 MHz) of 18 in CDCl_3_	49](#_Toc225599259)

[Figure S48. ^13^C{^1^H) NMR (125 MHz) of 18 in CDCl_3_	49](#_Toc225599260)

[Figure S49. ^1^H NMR (500 MHz) of 19 in CDCl_3_	50](#_Toc225599261)

[Figure S50. ^13^C{^1^H) NMR (125 MHz) of 19 in CDCl_3_	50](#_Toc225599262)

[Figure S51. ^1^H NMR (400 MHz) of 20 in CDCl_3_	51](#_Toc225599263)

[Figure S52. ^13^C{^1^H) NMR (125 MHz) of 20 in CDCl_3_	51](#_Toc225599264)

[Figure S53. ^1^H NMR (500 MHz) of 16 in CDCl_3_	52](#_Toc225599265)

[Figure S54. ^13^C{^1^H) NMR (125 MHz) of 16 in CDCl_3_	52](#_Toc225599266)

[Figure S55. ^1^H-^1^H COSY NMR (500 MHz) 16 in CDCl_3_	53](#_Toc225599267)

[Figure S56. ^1^H-^1^H NOESY NMR (500 MHz) 16 in CDCl_3_	54](#_Toc225599268)

[Figure S57. ^1^H-^13^C HSQC NMR (500 MHz) 16 in CDCl_3_	55](#_Toc225599269)

[Figure S58. ^1^H-^13^C HMBC NMR (500 MHz) 16 in CDCl_3_	56](#_Toc225599270)

[Table S1. Assignment of ^1^H NMR peaks of **16** through combination of ^1^H-^1^H COSY and NOESY.	57](#_Toc225599271)

[Table S2. Assignment of ^13^C NMR peaks of **16** through combination of ^1^H-^13^C HSQC and HMBC.	58](#_Toc225599272)

[4.	IR spectra	59](#_Toc225599273)

[Figure S59. FT-IR of [7](OTf)_2_	59](#_Toc225599274)

[Figure S60. FT-IR of [8](OTf)	59](#_Toc225599275)

[5.	HRMS spectra	60](#_Toc225599276)

[Figure S61. HRMS of [1](BF_4_)	60](#_Toc225599277)

[Figure S62. HRMS of [2](OTf)	60](#_Toc225599278)

[Figure S63. HRMS of [2](BF_4_)	61](#_Toc225599279)

[Figure S64. HRMS of [3](BF_4_)	61](#_Toc225599280)

[Figure S65. HRMS of [4](OTf)	61](#_Toc225599281)

[Figure S66. HRMS of [5](OTf)	62](#_Toc225599282)

[Figure S67. HRMS of [6](BF_4_)_2_	62](#_Toc225599283)

[Figure S68. HRMS of [7](OTf)_2_	63](#_Toc225599284)

[Figure S69. HRMS of [8](OTf)	63](#_Toc225599285)

[Figure S70. HRMS of 15	64](#_Toc225599286)

[Figure S71. HRMS of 16	65](#_Toc225599287)

[Figure S72. HRMS of 18	65](#_Toc225599288)

[Figure S73. HRMS of 17	66](#_Toc225599289)

[Figure S74. HRMS of 19	67](#_Toc225599290)

[Figure S75. HRMS of 20	68](#_Toc225599291)

[6.	Crystallography	69](#_Toc225599292)

[6.1.	General	69](#_Toc225599293)

[Table S3. CCDC deposition numbers of XRD structures.	69](#_Toc225599294)

[6.2.	Supplementary XRD structures	69](#_Toc225599295)

[Figure S76. XRD structure of complex [8](OTf)	69](#_Toc225599296)

[Figure S77. XRD structure of compound 19	70](#_Toc225599297)

[6.3.	Crystallographic parameters.	70](#_Toc225599298)

[Table S4. Selected bond lengths and bond angles Rh(I) sulfonium complexes **[3]**(BF_4_), **[4]**(OTf), **[5]**(OTf), and **[6]**(BF_4_)_2_	70](#_Toc225599299)

[Table S5. Crystallographic parameters of compounds **[2]**(OTf), **[3]**(BF_4_), **[4]**(OTf), and **[5]**(OTf).	71](#_Toc225599300)

[Table S6. Crystallographic parameters of compounds **[6]**(BF_4_)_2_, **[7]**(OTf)_2_, **[8]**(OTf), and **19**	72](#_Toc225599301)

[7.	Computational data	73](#_Toc225599302)

[7.1.	Input templates	73](#_Toc225599303)

[7.2.	Summary of the computational results	73](#_Toc225599304)

[Figure S78. Model structures of isostructural series of Rh(I) complexes of coordinated acetone molecule with Si-, P-, and S-based tripodal ligands used for computational studies	73](#_Toc225599305)

[Table S7. Selected bond lengths and angles in optimized model structures **10***, **8***, and **7*** compared to those in the XRD structures of complexes **10**, **[8]**(OTf), and **[7]**(OTf)_2_	73](#_Toc225599306)

[Table S8. Selected bond lengths and angles in the optimized model complexes **21***, **22*** and **23***.	74](#_Toc225599307)

[Table S9. Natural Population charges of selected atoms in Rh(CO) and Rh(Acetone) complexes.	74](#_Toc225599308)

[Table S10. Selected bond Wiberg indices	74](#_Toc225599309)

[Table S11. NLMOs representing the major E-Rh bonding interaction in the optimized model complexes **10***, **8*** and **7*** (isovalue = 0.03).	75](#_Toc225599310)

[Table S12. NLMOs representing the major E-Rh bonding interaction in the optimized model complexes **21***, **22*** and **23*** (isovalue = 0.03).	75](#_Toc225599311)

[8.	Additional References	76](#_Toc225599312)

# 1. General information

All air sensitive reactions were carried out under an atmosphere of purified nitrogen in a glovebox equipped with an inert gas purifier. Tetrahydrofuran (THF), acetonitrile (MeCN), diethyl ether (Et_2_O), dichloromethane (DCM) and toluene were purified by passing through a column of activated alumina under inert atmosphere. TlBF_4_,^[74]^ [Rh(Me)(COD)]_2_,^[75]^ RhH(PPh_3_)_3_,^[76]^ were prepared according to the literature procedures. KPPh_2_, AgBF_4_, NaBF_4_, [RhCl(COD)]_2_, [RhCl(CO)_2_]_2_, [Rh(COD)_2_]BF_4_, TPPO, TEPO, o-DFB, methylindole, benzofuran, acetone, 3-pentanone, cyclohexanone, acetophenone and benzaldehyde were used as purchased. Anhydrous n-hexane, n-pentane, chloroform (CHCl_3_) and benzene packed under inert gas over molecular sieves were used as purchased. All chromatographic purifications were performed on CombiFlash EZ-Prep and SiO_2_ columns, this allowed fast purification of phosphine-containing ligands under air without noticeable oxidation. NMR spectra were recorded on Bruker Avance 400 or Bruker Avance 500 spectrometers at 296K. Residual solvent peaks were used as internal standards for ^1^H (CDCl_3_: δ 7.26 ppm; CD_2_Cl_2_: δ 5.31 ppm, acetone-d_6_: δ 2.05 ppm, THF-d_8_: δ 3.58, 1.72 ppm, and CD_3_CN: δ 1.94 ppm, C_6_D_4_Cl_2_: δ 7.19, 6.93 ppm). Other nuclei were referenced to the ^1^H reference frequency multiplied by the standard ratios: ^[77]^ ^13^C{^1^H} 0.25145020, ^19^F 0.94094011 and ^31^P{^1^H} 0.40480742. NMR data are reported as follows: chemical shift, multiplicity (s = singlet, d = doublet, t = triplet, q = quartet, m = multiplet, br = broad, coupling constant(s), and integration. High resolution mass spectra (HRMS) were recorded on HR Q-TOF-MS mass instrument, using an electrospray ionization (ESI+) technique (MeCN/H_2_O 80%, flow: 0.2 ml/min). FT-IR spectra were measured using Bruker AlPHA II compact spectrometer. Elemental analysis was performed by Mikroanalytisches Laboratorium Kolbe (Germany).

Electrochemical experiments were performed at room temperature under N_2_ atmosphere using μAutolab type II potentiometer. The electrochemical cell used consisted of a low-volume three electrodes cell setup with glassy carbon working electrode (3 mm diameter), platinum counter/auxiliary electrode and Ag/AgNO_3_ (0.01M in 0.1 M tetra-butyl ammonium hexafluorophosphate in acetonitrile) reference electrode (Pine research instrumentation). 0.1 M tetrabutylammonium hexafluorophosphate has been used as a supporting electrolyte.

The calculations were performed using Gaussian 16, Revision C.01.^[78]^ Geometry optimization and frequency calculations for all presented structures were carried out with the ωB97XD^[79]^ functional and the def2TZVP^[80]^ basis set. The optimized structures were confirmed through vibrational frequency analysis, where all considered structures showed no imaginary frequencies. The coordinates of the optimized geometries are provided in a separate file. Natural Population Analysis were carried out with the Natural Bond Orbital (NBO 7.0)^[81]^ program at the same level of theory as optimization of the structures.

# 2. Synthetic procedures

## 2.1 Preparation of tripodal sulfonium ligands

*Bis(2-(diphenylphosphaneyl)phenyl)(2-fluorophenyl)sulfonium tetrafluoroborate,* ***[1]****(BF_4_).*

Compound **[1]**(BPh_4_) (1.58 g, 1.64 mmol) was dissolved in 130 ml of MeCN. TlBF_4_ (476.4 mg, 1.0 eq.) was added as solid to the solution. Immediately after adding the TlBF_4_ the solution turned into a white emulsion that was stirred for 2 hours. The TlBPh_4_ precipitate that formed was collected by filtration, washed with MeCN several times, and discarded. The combined filtrate was evaporated in vacuo, dissolved in a minimal amount of DCM then precipitated with Et_2_O and evaporated to afford 1.20 g of the product as white solid (quantitative yield).

^1^H NMR (500 MHz, CD_3_CN): 7.76-7.70 (m, 3H), 7.57 (td, 2H, *J* = 7.8, 1.2 Hz), 7.45-7.30 (m, 15H), 7.28-7.13 (m, 11H), 7.06 (td, 1H, *J* = 7.5, 1.3 Hz) ppm. ^13^C{^1^H} NMR (125 MHz, CD_3_CN): 160.1 (d, ArF, *J*_C-F_ = 255.0 Hz), 140.8 (d, *J* = 16.5 Hz), 137.7 (d, *J* = 9.2 Hz), 137.0 (s), 134.7 (s), 133.6 (d, *J*_C-P_ = 4.6 Hz), 133.5 (d, *J*_C-P_ = 4.5 Hz), 133.4 (d, *J*_C-P_ = 4.6 Hz), 133.1 (s), 132.7 (d, *J*_C-P_ = 4.5 Hz), 132.3 (d, *J*_C-P_ = 5.9 Hz), 131.9 (t, *J* = 3.6 Hz), 130.0 (d, *J* = 1.8 Hz), 129.7 (d, *J* = 5.7 Hz), 129.4 (d, *J* = 5.7 Hz), 129.0 (q, *J* = 3.7 Hz), 127.4 (d, *J* = 3.6 Hz), 118.0 (d, *J* = 19.9 Hz), 110.7 (dt, *J* = 7.4 Hz) ppm. ^19^F NMR (376 MHz, CDCl_3_): -105.67 (q, ArF, *J* = 7.0 Hz), -151.42 (s, ^10^BF_4_), -151.47 (s, ^11^BF_4_) ppm. ^31^P{^1^H} NMR (162 MHz, CDCl_3_): -13.02 ppm (s) ppm. HRMS: calcd for [M]^+^, C_42_H_32_FP_2_S^+^: 649.1679; found: 649.1671. EA: calcd for C_42_H_32_BF_5_P_2_S: C 68.49, H 4.38; found C 68.17, H 4.31.

*Tris(2-(diphenylphosphaneyl)phenyl)sulfonium trifluoromethanesulfonate,* ***[2]****(OTf).*

In a round bottom flask equipped with a stirring bar, bis(2-(diphenylphosphaneyl)phenyl)(2-fluorophenyl)sulfonium trifluoromethane-sulfonate **[1]**(OTf) (200 mg, 250.4 µmol) was dissolved in THF (39 ml) and this solution (6.42 mM) was cooled to -30 °C in a freezer. Next, 0.45 ml of a 0.5 M solution of KPPh_2_ in THF (1.00 eq.) was diluted with additional THF up to 1.0 ml (approx. 0.225 M) inside a 1 ml syringe that was placed in a freezer as well. After cooling, the RB flask and the syringe were taken out of the freezer and the KPPh_2_ solution was added cautiously dropwise to a vigorously stirred solution of **[1]**(OTf) (into the vortex) at a rate of one droplet every 15 seconds with the red-orange color of KPPh_2_ disappearing immediately when added to the solution. After addition of ca. 1/3 of the KPPh_2_ solution, both the reaction solution and syringe with KPPh_2_ were cooled down again by placed them back in the freezer and the dropwise addition was continued second third of the KPPh_2_ solution was continued (the second 1/3 of the KPPh_2_ solution). The remaining 1/3 was added in a similar cooling/dropwise addition cycle. Full consumption of **[1]**(OTf) was verified by ^31^P{^1^H} NMR. Additional diluted solution of KPPh_2_ was added in case of an incomplete conversion. Finally, the resulting light-yellow solution was evaporated, affording the crude product as a light yellow solid. The product was purified by an automatic flash chromatography on a silica-gel eluting with acetone/CHCl_3_ (35:65%) affording the product, as a white solid (168 mg, 70% yield). XRD quality crystals of **[2]**(OTf) were obtained by a layer diffusion of n-hexane into a concentrated DCM solution.

Due to dynamic equilibrium resulting in a significant peak broadening at RT, ^1^H NMR spectrum of **[2]**(OTf) was also recorded at -90.0±1.00 °C.

^1^H NMR (500 MHz, acetone-d_6_): 7.75 (td, 3H, *J* = 7.5, 1.1 Hz), 7.58 (td, 3H, *J* = 7.7, 1.3 Hz) 7.56-7.41 (m, 12H), 7.41-7.25 (br, 12H), 7.25-7.15 (m, 8H), 7.04-6.91 (br, 4H). ^1^H NMR (500 MHz, acetone-d_6_, -90.0°C): 7.85 (t, 3H, *J* = 7.6 Hz), 7.69 (t, 3H, *J* = 7.6 Hz), 7.64-7.58 (m, 9H), 7.45 (dd, 3H, *J* = 7.6, 3.3 Hz), 7.37 (t, 3H, *J* = 7.35 Hz), 7.34-7.25 (m, 15H), 6.96 (t, 6H, *J* = 7.9 Hz) ppm. ^13^C{^1^H} NMR (125MHz, acetone-d_6_): 140.8 (d, *J*_C-P_ = 16.5 Hz), 137.0 (s), 134.2 (s), 133.7 (bs), 133.5 (bs), 132.7 (s), 132.6-132.5 (m), 131.9 (dt, *J*_C-P_ = 34.6, 7.3 Hz), 129.8 (bs), 129.0 (bs), 121.6 (q, OTf, *J*_C-F_ = 322.8 Hz) ppm. ^19^F NMR (470 MHz, acetone-d_6_): -78.7 (s, OTf) ppm. ^31^P{^1^H} NMR (202 MHz, acetone-d_6_): -14.32 (s) ppm. HRMS: calcd for [M]^+^, C_54_H_42_P_3_S^+^: 815.2220; found: 815.2157. EA: calcd for C_55_H_42_F_3_O_3_P_3_S_2_: C 68.46 ,H 4.39; found C 68.33, H 4.38.

*Tris(2-(diphenylphosphaneyl)phenyl)sulfonium tetrafluoroborate,* ***[2]****(BF_4_).*

This compound was prepared similarly to **[2]**(OTf) starting with a bis(2-(diphenylphosphaneyl)phenyl)(2-fluorophenyl)sulfonium tetrafluoroborate **[1]**(BF_4_) (200 mg, 250 µmol), affording the product **[2]**(BF_4_) as a white solid (146 mg, 57% yield).

^1^H NMR (500 MHz, acetone-d_6_): 7.75 (td, 3H, *J* = 7.5, 1.0 Hz), 7.58 (t, 3H, *J* = 7.7 Hz) 7.56-7.41 (m, 12H), 7.41-7.25 (m, 12H), 7.25-7.13 (m, 8H), 7.04-6.90 (m, 4H) ppm. ^13^C{^1^H} NMR (125MHz, acetone-d_6_): 140.8 (d, *J* = 17.0 Hz), 137.0 (s), 134.2 (s), 133.7 (bs), 133.6 (bs), 132.6 (s), 132.5-132.4 (m), 131.9 (dt, *J* = 36.1, 7.2 Hz), 129.8 (bs), 129.0 (bs) ppm. ^19^F NMR (376 MHz, acetone-d6): -151.74 (s, ^10^BF_4_), -150.79 (s, ^11^BF_4_) ppm. ^31^P{^1^H} NMR (162 MHz, acetone-d_6_): -14.35 (s) ppm. HRMS: calcd for [M]^+^, C_54_H_42_P_3_S^+^: 815.2220; found: 815.2181. EA: calcd for C_54_H_42_BF_4_P_3_S: C 71.85, H 4.69; found C 71.55, H 4.70.

## 2.2 Preparation of Rh(I) complexes of tripodal sulfonium and phosphine ligands

*Tris(2-(diphenylphosphaneyl)phenyl)sulfonium chlororhodium(I) tetrafluoroborate,* ***[3]****(BF_4_).*

Ligand **[2]**(BF_4_) (40 mg, 44.3 µmol) was placed in a glass vial and suspended in toluene (4.0 ml). In a separate vial [RhCl(COD)]_2_ (10.2 mg, 0.5 eq.) was dissolved in toluene (5.0 ml) and added to a stirred suspension of **[2]**(BF_4_). The vial was capped and left to stir overnight during which time a dark-purple precipitate gradually formed. The solvent was removed, and the solid residue was first washed with toluene until the filtrate was colorless, then a few times with pentane and finally dried under vacuum to afford the product as a dark purple powder (39 mg, 85% yield). XRD quality crystals of **[3]**(BF_4_) were then obtained by layer diffusion of n-hexane into a its concentrated solution in CHCl_3_ at -30 °C.

^1^H NMR (500 MHz, CDCl_3_): 9.27 (d, 3H, *J* = 8.3 Hz), 8.16 (t, 3H, *J* = 7.7 Hz), 7.72 (t, 3H, *J* = 7.5 Hz), 7.66-7.63 (m, 3H), 7.22 (t, 6H, *J* = 7.0 Hz), 7.06-6.98 (m, 24H) ppm. ^13^C{^1^H} NMR (125 MHz, CDCl_3_(: 141.5-141.0 (m), 137.1 (dd, *J* = 22.1, 8.0 Hz), 135.8 (s), 135.1 (s), 134.5 (s), 134.2-133.7 (m), 133.2 (d, *J* = 8.2 Hz), 132.2 (s), 129.8 (broad s), 129.6 (s), 128.5 (s) ppm. ^19^F NMR (376 MHz, CDCl_3_): -152.72 (s, ^10^BF_4_), -152.78 (s, ^11^BF_4_) ppm. ^31^P{^1^H} NMR (162 MHz, CDCl_3_): 41.5 (d, ^1^*J*_P-Rh_ = 130.5 Hz) ppm. HRMS: calcd for [M]^+^, C_54_H_42_ClP_3_RhS^+^: 953.0958; found: 953.0927. EA: calcd for C_54_H_42_BClF_4_P_3_RhS: C 62.30, H 4.07; found C 61.15, H 4.11. The lower carbon content is most probably due to an incomplete removal of the 3 CHCl_3_ molecules per unit formula present in the crystal lattice. Compare, for instance, calcd for C_54_H_42_BClF_4_P_3_RhS(CHCl_3_)_0.15_: C 61.32, H 4.01; found C 61.15, H 4.11.

*Tris(2-(diphenylphosphaneyl)phenyl)sulfonium rhodium(I) methyl trifluoromethanesulfonate,* ***[4]****(OTf).*

Ligand **[2]**(OTf) (30 mg, 31.1 µmol) was placed in a glass vial and partially dissolved in toluene (5.3 ml). In an additional vial Bis[(1,2,5,6-η)-1,5-cyclooctadiene]di-μ-methyldirhodium [Rh(Me)(COD)]_2_ (7.0 mg, 0.5 eq.) was dissolved in toluene as well (1.0 ml) and added to the vial containing the solution of **[2]**(OTf). The vial was capped, left to stir overnight and brown precipitate formed gradually. The solvent was removed, the solid was washed with some toluene, later with n-pentane and dried under vacuum at -30 °C to obtain the product as light-brown powder. (30.3 mg, 90% yield). XRD quality crystals were then obtained by layer diffusion of n-pentane into a concentrated CHCl_3_ solution at -30 °C.

Due to the low stability of **[4]**(OTf) in solution at r.t. for a prolonged time, its ^1^H, ^13^C and 2D NMR spectra were recorded at -30.0±1.00 °C.

^1^H NMR (500 MHz, CDCl_3_, -30 °C): 9.49 (d, 3H, *J* = 8.3 Hz), 8.08 (t, 3H, *J* = 7.7 Hz), 7.65 (t, 3H, *J* = 7.5 Hz), 7.57-7.54 (m, 3H), 7.23 (t, 6H, *J* = 7.4 Hz), 7.01 (t, 12H, *J* = 7.7 Hz), 6.88-6.83 (m, 12H), 1.17 (dq, 3H, ^3^*J*_H-P_ = 9.3 Hz, ^2^*J*_H-Rh_ = 1.6 Hz MeRh) ppm. ^13^C{^1^H} NMR (125 MHz, CDCl_3_, -30 °C): 140.8 (dd, *J* = 16, 3.8 Hz), 140.0 (dd, *J* = 18.2, 8.6 Hz), 135.9-135.6 (m), 135.5 (s), 134.1 (s), 133.5 (s), 131.7 (dd, *J* = 9.9, 5 Hz), 129.7 (broad s), 129.1 (s), 128.6 (s), 120.9 (q, OTf, *J* = 320 Hz), -10.8 (dq, MeRh, ^1^*J*_C-Rh_ = 18.7, ^2^*J*_C-P_ = 7.2 Hz) ppm. ^19^F NMR (376 MHz, CDCl_3_): -77.93 (s, OTf) ppm. ^31^P{^1^H} NMR (162 MHz, CDCl_3_): 45.0 (d, ^1^*J*_P-Rh_ = 159.1 Hz) ppm. HRMS: calcd for [M]^+^, C_55_H_45_P_3_RhS^+^: 933.1504; found: 933.1477. Due to poor thermal stability reliable EA data could not be obtained.

*Tris(2-(diphenylphosphaneyl)phenyl)sulfonium rhodium(I) hydride trifluoromethanesulfonate,* ***[5]****(OTf).*

Ligand **[2]**(OTf) (40 mg, 41.5 µmol) was placed in a glass vial and partially dissolved in toluene (3.3 ml). In an additional vial tris(triphenylphosphine)rhodium(I) hydride RhH(PPh_3_)_3_ (36.9 mg, 1.0 eq.) was dissolved as well in toluene (3.3 ml) and added to the vial containing the solution of **[2]**(OTf). The vial was capped, left to stir overnight and orange precipitate formed gradually. The solvent was removed, the solid was washed consecutively with some toluene, benzene, 1,4-dioxane and n-pentane, dried under vacuum to obtain the product as orange powder. (35.4 mg, 80% yield). XRD quality crystals were then obtained by layer diffusion of n-hexane into a concentrated benzene solution.

^1^H NMR (500 MHz, THF-d_8_): 9.96 (d, 3H, *J* = 8.3 Hz), 8.13 (t, 3H, *J* = 7.8 Hz), 7.75 (t, 3H, *J* = 7.4 Hz), 7.69-7.66 (m, 3H), 7.11 (t, 6H, *J* = 7.2 Hz), 6.98-6.94 (m, 12H), 6.69 (t, 12H, *J* = 7.4 Hz) -6.36 (dq, 1H, ^1^*J*_H-Rh_ = 11.8, ^2^*J*_H-P_ = 7.4 Hz) ppm. ^13^C{^1^H} NMR (125 MHz, THF-d_8_ (: 142.2-141.7 (m), 140.9-140.6 (m), 137.1-136.6 (m), 135.9 (s), 133.9 (s), 133.6 (s), 132.9-132.7 (m), 131.9-131.7 (m), 130.6 (broad s), 128.6 (s), 128.1 (broad s), 121.1 (q, OTf, *J* = 321 Hz) ppm. ^19^F NMR (376 MHz, CDCl_3_): -79.30 (s, OTf) ppm. ^31^P{^1^H} NMR (162 MHz, THF-d_8_): 61.52 (d, ^1^*J*_P-Rh_ = 156.7 Hz) ppm. HRMS: calcd for [M]^+^, C_54_H_43_P_3_RhS^+^: 919.1348; found: 919.1325. EA: calcd for C_55_H_43_F_3_O_3_P_3_RhS_2_: C: 61.80, H: 4.06; found for C: 61.02, H: 4.28.

*Tris(2-(diphenylphosphaneyl)phenyl)sulfonium rhodium(I) acetonitrile Bis(tetrafluoroborate),* ***[6]****(BF_4_)_2_.*

Ligand **[3]**(BF_4_) (20 mg, 19.2 µmol) was dissolved in MeCN (2.0 ml) and the dark purple solution was added to a vial containing AgBF_4_ (3.7 mg, 1.0 eq.) and a stirring bar. Gradually the solution turned dark red. After 15 min. the solution was filtered through Celite to obtain a clear dark red solution that was dried under vacuum to obtain the product as a dark red powder (21.8 mg, quantitative yield). XRD quality crystals of **[6]**(BF_4_)_2_ were then obtained by layer diffusion of Et_2_O into a its concentrated solution in MeCN at -30 °C.

^1^H NMR (500 MHz, CD_3_CN): 8.64 (d, 3H, *J* = 8.2 Hz), 8.02 (t, 3H, *J* = 7.8 Hz), 7.91 (t, 3H, *J* = 7.5 Hz), 7.84-7.80 (m, 3H), 7.39 (t, 6H, *J* = 7.5 Hz), 7.19 (t, 12H, *J* = 7.6 Hz), 7.06-7.01 (m, 12H) ppm. ^13^C{^1^H} NMR (125 MHz, CD_3_CN (: 138.4 (ddd, *J* = 28.4, 4.2, 3.4 Hz), 136.5-136.2 (m), 136.2 (s), 135.9 (s), 134.2 (s), 132.1 (dd, *J* = 9.8, 5.0 Hz), 131.9 (dd, *J* = 14.2, 4.1 Hz), 130.9 (s), 129.7 (dd, *J* = 9.0, 4.5 Hz), 129.2 (q, *J* = 3.4 Hz) ppm. ^19^F NMR (470 MHz, CD_3_CN): -151.76 (s, ^10^BF_4_), -151.81 (s, ^11^BF_4_) ppm. ^31^P{^1^H} NMR (202 MHz, CD_3_CN): 46.16 (d, ^1^*J*_P-Rh_ = 123.6 Hz) ppm. HRMS: calcd for [M-CH_3_CN]^2+^, C_54_H_42_P_3_RhS^2+^: 459.0638 (double charge); found: 459.0636. EA: calcd for C_56_H_45_B_2_F_8_NP_3_RhS: C: 59.34 ,H: 4.00, N: 1.24; found for C: 58.67, H: 3.90, N: 1.21.

*Tris(2-(diphenylphosphaneyl)phenyl)sulfonium rhodium(I) carbonyl bis(trifluoromethanesulfonate),* ***[7]****(OTf)_2_.*

Ligand **[2]**(OTf) (40 mg, 41.5 µmol) and NaOTf (7.7 mg, 1.0 eq.) were placed in a glass vial and dissolved in 1.1 ml of THF. In a separate vial [RhCl(CO)_2_]_2_ (8.1 mg, 0.5 eq.) was dissolved in additional 1.1 ml of THF and added to the first solution. Upon the addition of [RhCl(CO)_2_]_2_ the solution gradually turned dark orange. The vial was capped, left to stir overnight during which time an orange precipitate slowly formed. The vial was kept at -30 °C overnight, the solvent was decanted by syringe, and the solid was washed with a small amount of a precooled (-30 °C) THF and followed by Et_2_O, until the filtrate became colorless, the orange precipitate was dried under vacuum. The precipitate was then dissolved in DCM, filtered through Celite to obtain a clear red solution that was precipitated with Et_2_O and dried under vacuum to obtain the product as an orange powder. (38.3 mg, 84% yield). XRD quality crystals of **[7]**(OTf)_2_ were obtained by a layer diffusion of Et_2_O into its concentrated solution in CHCl_3_.

^1^H NMR (400 MHz, CDCl_3_): 9.52 (d, 3H, *J* = 8.4 Hz), 8.35 (t, 3H, *J* = 7.8 Hz) 8.04 (t, 3H, *J* = 7.6 Hz), 7.77-7.72 (m, 3H), 7.42 (t, 6H, *J* = 7.4 Hz), 7.19 (t, 12H, *J* = 7.6 Hz), 6.91-6.85 (m, 12H) ppm. ^13^C{^1^H} NMR (125 MHz, CDCl_3_): 193.6 (dq, carbonyl, *J* = 62.1 ,11.9 Hz), 137.4 (s), 137.2-136.9 (m), 136.8 (s), 136.3 (s), 135.1 (q, *J* = 12.4 Hz), 132.2 (s), 130.3 (dd, *J* = 16.8, 7.6 Hz), 129.7 (s), 128.6 (s), 120.9 (q, OTf, *J* = 321.8 Hz) ppm. ^19^F NMR (470 MHz, CD_2_Cl_2_): -78.79 (s, OTf) ppm. ^31^P{^1^H} NMR (202 MHz, CD_2_Cl_2_): 57.07 (d, ^1^*J*_P-Rh_ = 117.7 Hz) ppm. FT-IR: ν(Rh-C≡O) 2030 (s) cm^-1^. HRMS: calcd for [M]^2+^, C_55_H_42_OP_3_RhS^2+^: 473.0607 (double charge); found: 473.0606. EA: calcd for C_57_H_42_F_6_O_7_P_3_RhS_3_: C: 54.99 ,H: 3.40; found for C: 54.80, H: 3.38.

*Tris(2-(diphenylphosphaneyl)phenyl)phosphine rhodium(I) carbonyl trifluoromethanesulfonate,* ***[8]****(OTf).*

Ligand **9** (40 mg, 41.5 µmol) and NaOTf (7.7 mg, 1.0 eq.) were placed in a glass vial and dissolved in 1.3 ml of THF. In a separate vial RhCl(CO)_2_]_2_ (9.5 mg, 0.5 eq.) was dissolved in 1.3 ml THF and added to the first solution. Upon the addition of [RhCl(CO)_2_]_2_ the solution turned orange red. The vial was capped, left to stir overnight. The solvent was then removed under vacuum, and the solid residue was dissolved in acetone and filtered through Celite. The filtrate volume was then reduced to a minimum, after which Et_2_O was added to precipitate the product as a yellow powder that was then dried under vacuum. (47.2 mg, 88% yield). XRD quality crystals of **[8]**(OTf) were obtained by a layer diffusion of Benzene into its concentrated solution in DCM.

^1^H NMR (500 MHz, CD_2_Cl_2_): 8.63 (t, 3H, *J* = 7.7 Hz), 7.85 (t, 3H, *J* = 7.2 Hz), 7.6 (t, 3H, *J* = 7.2 Hz), 7.49-7.44 (m, 3H), 7.26 (t, 6H, *J* = 7.3 Hz), 7.0 (t, 12H, *J* = 7.6 Hz), 6.91 (dd, 12H, *J* = 11, 7.1 Hz) ppm. ^13^C{^1^H} NMR (125 MHz, CD_2_Cl_2_): 201.2 (ddq, carbonyl, *J* = 99.7, 54.3, 12.5 Hz), 142.9-142.2 (m), 140.3-139.3 (m), 135.5-135.0 (m), 133.8 (d, *J* = 17.8 Hz), 132.6 (s), 132.2 (d, *J* = 6.9 Hz), 131.6 (dd, *J* = 9.8, 5.0 Hz), 129.8 (s), 128.7 (q, *J* = 3.4 Hz), 121.2 (q, OTf, *J* = 321.8 Hz) ppm. ^19^F NMR (376 MHz, CD_2_Cl_2_): -78.82 (s, OTf) ppm. ^31^P{^1^H} NMR (162 MHz, CD_2_Cl_2_): 122.42 (dq, 1P, ^1^*J*_P(A)-Rh_ = 88.0, ^3^*J*_P(A)-P(B)_ = 26.9 Hz), 55.8 (dd, 3P, ^1^*J*_P(B)-Rh_ = 131.7, ^3^*J*_P(B)-P(A)_ = 26.9 Hz) ppm. FT-IR: FT-IR: ν(Rh-C≡O) 1986s (CO) cm^-1^. HRMS: calcd for [M]^+^, C_55_H_42_OP_4_Rh^+^: 945.1236; found: 945.1195. EA: calcd for C_56_H_42_F_3_O_4_P_4_RhS: C: 61.44 ,H: 3.87; found for C: 61.15, H: 3.84.

## 2.3 Gutmann-Beckett method measurements of cationic Rh(I) sulfonium and phosphine complexes

**[2]**(BF_4_) (5 mg, 5.54 µmol) or **9** (5 mg, 6.14 µmol) and bis(1,5-cyclooctadiene)rhodium(I) tetrafluoroborate, [Rh(COD)_2_]BF_4_ (2.2 mg or 2.5 mg, respectively, 1 eq.), were added to a J-Young tube and o-DFB (0.4 ml, containing TMS) was added, the solution obtained a dark purple or dark red (respectively) color, the tube was closed and mixed several times. After approx. 30 min, the reaction was checked by ^31^P{^1^H} NMR, validating no free ligand is present. Triphenylphosphine oxide TPPO (1.5 mg, or 1.5 mg, respectively, 1 eq.) was added to the J-young tube, the tube was closed and mixed again before measuring ^31^P{^1^H} NMR to establish the Rh^+^---TPPO interaction. The experiments with TEPO (0.7 mg or 0.8 mg, respectively, 1 eq.) were carried out in a similar manner.

## 2.4 Optimized procedure for the catalytic reaction

Inside glovebox, ligand **[2]**(BF_4_) (13.4 mg, 3 mol%) was added to a pressure tube equipped with a stirring bar and dissolved in 0.5 ml of the designated ketone. Next [Rh(COD)_2_]BF_4_ (6 mg, 3 mol%) was added as a solid. The vial in which it was weighed was washed with additional 0.5 ml of the designated ketone, and the washing solution was added to the reaction tube. The nucleophile (0.5 mmol) was then added, the tube was capped, taken out of the glovebox and heated to 50 °C for 12 hours. After cooling to RT, the solvent was evaporated and the product was purified by a flash chromatography on a silica gel, as specified below, resulting in the previously reported compounds **15**,^[82]^ **17**,^[82]^ **18**,^[83]^ **19**,^[82]^ and **20**.^[84]^

**3,3'-(pentane-3,3-diyl)bis(1-methyl-1H-indole)**, **15**.

Purified by flash column chromatography, 4.5% EtOAc in hexane, the title compound was isolated as a white solid (93% yield). ^1^H NMR (400 MHz, CDCl_3_): 7.26-7.22 (m, 4H), 7.07 (t, 2H, *J* = 7.6 Hz), 7.02 (s, 2H), 6.80-6.75 (m, 2H), 3.80 (s, 6H), 2.31 (q, 4H, *J* = 7.3 Hz), 0.69 (t, 6H, *J* = 7.3 Hz) ppm. ^13^C{^1^H} NMR (100 MHz, CDCl_3_): 137.7, 127.2, 126.9, 121.4, 121.3, 117.8, 108.7, 42.2, 32.7, 28.5, 8.5 ppm. HRMS: calcd for [M+H]^+^, C_23_H_26_N_2_^+^: 331.2169; found: 331.2162.

**1,1,3-triethyl-2,4-dimethyl-1,4-dihydrocyclopentaindole**, **16**.

Purified by flash column chromatography, 5% EtOAc in hexane, the title compound was isolated as a yellow oil (42% yield). ^1^H NMR (500 MHz, CDCl_3_): 7.49-7.45 (m, 1H), 7.35-7.30 (m, 1H), 7.12-7.07 (m, 2H), 3.90 (s, 3H), 2.64 (q, 2H, *J* = 7.5 Hz), 2.01 (dq, 2H, *J* = 7.4, 1.4 Hz), 1.81 (s, 3H), 1.60 (dq, 2H, *J* = 7.4, 1.4 Hz), 1.21 (t, 3H, *J* = 7.5 Hz), 0.45 (t, 6H, *J* = 7.4 Hz) ppm. ^13^C{^1^H} NMR (125 MHz, CDCl_3_): 148.1, 147.5, 140.8, 132.6, 123.9, 122.5, 118.9, 118.6, 117.5, 109.4, 54.6, 30.6, 29.3, 19.0, 14.7, 9.8, 8.6 ppm. HRMS: calcd for [M+H]^+^, C_19_H_26_N^+^: 268.2065; found: 268.2059.

**3,3'-(propane-2,2-diyl)bis(1-methyl-1H-indole)**, **17**.

Purified by flash column chromatography, 4.5% EtOAc in hexane, the title compound was isolated as a white solid (93% yield). ^1^H NMR (500 MHz, CDCl_3_): 7.45 (d, 2H, *J* = 8.1 Hz), 7.26 (d, 2H, *J* = 8.2 Hz), 7.12 (t, 2H, *J* = 7.6 Hz), 6.91-6.88 (m, 2H), 6.88 (s, 2H), 3.75 (s, 6H), 1.90 (s, 6H) ppm. ^13^C{^1^H} NMR (125 MHz, CDCl_3_): 137.8, 126.7, 125.5, 124.1, 121.5, 120.9, 118.1, 109.1, 35.0, 32.7, 30.4 ppm. HRMS: calcd for [M]^+^, C_21_H_22_N_2_^+^: 302.1783; found: 302.1773.

**3,3'-(cyclohexane-1,1-diyl)bis(1-methyl-1H-indole)**, **18**.

Purified by flash column chromatography, 4.5% EtOAc in hexane, the title compound was isolated as a white solid (98% yield). ^1^H NMR (500 MHz, CDCl_3_): 7.45 (dt, 2H, *J* = 8.1 ,0.9 Hz), 7.25 (d, 2H, *J* = 8.1 Hz), 7.15-7.11 (m, 2H), 6.95-6.92 (m, 2H), 6.91 (s, 2H), 3.74 (s, 6H), 2.58-2.55 (m, 4H), 1.70-1.65 (m, 4H), 1.63-1.58 (m, 2H) ppm. ^13^C{^1^H} NMR (125 MHz, CDCl_3_): 137.7, 127.0, 126.7, 122.3, 121.7, 120.7, 117.9, 109.1, 39.7, 37.1, 32.7, 26.8, 23.1 ppm. HRMS: calcd for [M]^+^, C_24_H_26_N_2_^+^: 342.2096; found: 342.2095.

**3,3'-(1-phenylethane-1,1-diyl)bis(1-methyl-1H-indole)**, **19**.

Purified by flash column chromatography, 5% EtOAc in hexane, the title compound was isolated as a white solid (96% yield). ^1^H NMR (500 MHz, CDCl_3_): 7.41-7.39 (m, 2H), 7.30 (t, 4H, *J* = 8.6 Hz), 7.27—7.23 (m, 3H), 7.21-7.15 (m, 3H), 6.95-6.91 (m, 2H), 6.48 (s, 2H), 3.68 (s, 6H), 2.35 (s, 3H) ppm. ^13^C{^1^H} NMR (125 MHz, CDCl_3_): 148.4, 137.8, 128.1, 127.8, 126.8, 125.7, 123.3, 122.2, 121.0, 118.4, 109.1, 43.7, 32.6, 29.1 ppm. HRMS: calcd for [M+H]^+^, C_26_H_25_N_2_^+^: 365.2012; found: 365.1993.

**3,3'-(phenylmethylene)bis(1-methyl-1H-indole)**, **20**.

Purified by flash column chromatography, 7.5% EtOAc in hexane, the title compound was isolated as a pink solid (94% yield). ^1^H NMR (400 MHz, CDCl_3_): 7.40-7.33 (m, 4H), 7.31-7.27 (m, 4H), 7.23 (m, 3H), 7.02-6.97 (m, 2H), 6.53 (s, 2H), 5.88 (s, 1H), 3.68 (s, 6H) ppm. ^13^C{^1^H} NMR (125 MHz, CDCl_3_): 144.5, 137.4, 128.7, 128.3, 128.2, 127.5, 126.0, 121.4, 120.0, 118.6, 118.3, 109.1, 40.1, 32.7 ppm. HRMS: calcd for [M+H]^+^, C_25_H_23_N_2_^+^: 351.1856; found: 351.1711.

# 3. NMR spectra

a.

b.

Figure S1. ^1^H NMR (500 MHz) of [1](BF_4_) in CD_3_CN. a) full spectrum, b) aromatic region expanded.

a.

b.

Figure S2. ^13^C{^1^H) NMR (125 MHz) of [1](BF_4_) in CD_3_CN**.** a) full spectrum, b) aromatic region expanded.

Figure S3. ^31^P{H} NMR (162 MHz) of [1](BF_4_) in CDCl_3_**.**

Figure S4. ^19^F NMR (471 MHz) of [1](BF_4_) in CDCl_3_**.**

a.

b.

Figure S5. ^1^H NMR (500 MHz) of [2](OTf) in acetone-d_6_. a) full spectrum, b) aromatic region expanded.

a.

b.

Figure S6. ^13^C{^1^H) NMR (125 MHz) of [2](OTf) in acetone-d_6_. a) full spectrum, b) aromatic region expanded.

Figure S7. ^31^P{H} NMR (202 MHz) of [2](OTf) in acetone-d_6_**.**

Figure S8. ^19^F NMR (471 MHz) of [2](OTf) in acetone-d_6_**.**

a.

b.

Figure S9. ^1^H NMR (500 MHz) of [2](OTf) in acetone-d_6_ at -90.0±1.00°C. a) full spectrum, b) aromatic region expanded.

a.

b.

Figure S10. ^1^H NMR (500 MHz) of [2](BF_4_) in acetone-d_6_. a) full spectrum, b) aromatic region expanded.

a.

b.

Figure S11. ^13^C{^1^H) NMR (125 MHz) of [2](BF_4_) in acetone-d_6_. a) full spectrum, b) aromatic region expanded.

Figure S12. ^31^P{H} NMR (162 MHz) of [2](BF_4_) in acetone-d_6_.

Figure S13. ^19^F NMR (376 MHz) of [2](BF_4_) in acetone-d_6_.

a.

b.

Figure S14. ^1^H NMR (500 MHz) of [3](BF_4_) in CDCl_3_. a) full spectrum, b) aromatic region expanded.

a.

b.

Figure S15. ^1^*^3^*C{^1^H) NMR (*125* MHz) of [3](BF_4_) in CDCl_3_. a) full spectrum, b) aromatic region expanded.

Figure S16. ^31^P{H} NMR (162 MHz) of [3](BF_4_) in CDCl_3_.

Figure S17. ^19^F NMR (376 MHz) of [3](BF_4_) in CDCl_3_.

Pentane

a.

b.

Figure S18. ^1^H NMR (500 MHz) of [4](OTf) in CDCl_3_ at -30.0±1.00 °C. a) full spectrum, b) aromatic region expanded.

Pentane

a.

b.

Figure S19. ^1^*^3^*C{^1^H) NMR (*125* MHz) of [4](OTf) in CDCl_3_ at -30.0±1.00 °C. a) full spectrum, b) aromatic region expanded.

Figure S20. ^31^P{H} NMR (162 MHz) of [4](OTf) in CDCl_3_.

Figure S21. ^19^F NMR (376 MHz) of [4](OTf) in CDCl_3_.

a.

b.

Figure S22. ^1^H NMR (500 MHz) of [5](OTf) in THF-d_8_. a) full spectrum, b) aromatic region expanded.

a.

b.

Figure S23. ^13^C{^1^H) NMR (125 MHz) of [5](OTf) in THF-d_8_. a) full spectrum, b) aromatic region expanded.

Figure S24. ^31^P{H} NMR (162 MHz) of [5](OTf) in THF-d_8_.

Figure S25. ^19^F NMR (376 MHz) of [5](OTf) in THF-d_8_.

a.

b.

Figure S26. ^1^H NMR (500 MHz) of [6](BF_4_)_2_ in CD_3_CN. a) full spectrum, b) aromatic region expanded.

a.

b.

Figure S27. ^1^*^3^*C{^1^H) NMR (125 MHz) of [6](BF_4_)_2_ in CD_3_CN. a) full spectrum, b) aromatic region expanded.

Figure S28. ^31^P{H} NMR (202 MHz) of [6](BF_4_)_2_ in CD_3_CN.

Figure S29. ^19^F NMR (470 MHz) of [6](BF_4_)_2_ in CD_3_CN.

a.

b.

Figure S30. ^1^H NMR (500 MHz) of [7](OTf)_2_ in CDCl_3_. a) full spectrum, b) aromatic region expanded.

a.

b.

Figure S31. ^13^C{^1^H) NMR (125 MHz) of [7](OTf)_2_ in CDCl_3_. a) full spectrum, b) aromatic region expanded.

Figure S32. ^31^P{H} NMR (202 MHz) of [7](OTf)_2_ in CD_2_Cl_2_.

Figure S33. ^19^F NMR (470 MHz) of [7](OTf)_2_ in CD_2_Cl_2_.

a.

b.

Figure S34. ^1^H NMR (500 MHz) of [8](OTf) in CD_2_Cl_2_. a) full spectrum, b) aromatic region expanded.

a.

b.

Figure S35. ^1^*^3^*C{^1^H) NMR (*125* MHz) of [8](OTf) in CD_2_Cl_2_. a) full spectrum, b) aromatic region expanded.

Figure S36. ^31^P{H} NMR (162 MHz) of [8](OTf) in CD_2_Cl_2_.

Figure S37. ^19^F NMR (376 MHz) of [8](OTf) in CD_2_Cl_2_.

-90 °C

-60 °C

-30 °C

0 °C

30 °C

50 °C

Figure S38. Varianable temperature ^1^H NMR (500 MHz) of [2](OTf) in acetone-d_6_.

Figure S39. ^1^H-^1^H COSY NMR (500 MHz) [2](OTf) in acetone-d_6_ at -40.0±1.00°C.

Figure S40. ^1^H-^13^C HSQC NMR (500 MHz) of [4](OTf) in CDCl_3_ at -30.0±1.00°C.

Figure S41. ^31^P{H} NMR (202 MHz) of [11](BF_4_)_2_ from a reaction between [3](BF_4_) and AgBF_4_ in C_6_D_4_Cl_2_. (W_1/2_ = 250 Hz).

a.

b.

Figure S42. ^1^H NMR (500 MHz) of [11](BF_4_)_2_ from a reaction between [3](BF_4_) and AgBF_4_ in C_6_D_4_Cl_2_.

Due to the poor stability of this compound ^13^C NMR spectrum could not be acquired.

Figure S43. ^1^H NMR (400 MHz) of 15 in CDCl_3_.

Figure S44. ^13^C{^1^H) NMR (100 MHz) of 15 in CDCl_3_.

Figure S45. ^1^H NMR (500 MHz) of 17 in CDCl_3_.

Figure S46. ^13^C{^1^H) NMR (125 MHz) of 17 in CDCl_3_.

Figure S47. ^1^H NMR (500 MHz) of 18 in CDCl_3_.

Figure S48. ^13^C{^1^H) NMR (125 MHz) of 18 in CDCl_3_.

Figure S49. ^1^H NMR (500 MHz) of 19 in CDCl_3_.

Figure S50. ^13^C{^1^H) NMR (125 MHz) of 19 in CDCl_3_.

Figure S51. ^1^H NMR (400 MHz) of 20 in CDCl_3_.

Figure S52. ^13^C{^1^H) NMR (125 MHz) of 20 in CDCl_3_.

Figure S53. ^1^H NMR (500 MHz) of 16 in CDCl_3_.

Figure S54. ^13^C{^1^H) NMR (125 MHz) of 16 in CDCl_3_.

Figure S55. ^1^H-^1^H COSY NMR (500 MHz) 16 in CDCl_3_.

Figure S56. ^1^H-^1^H NOESY NMR (500 MHz) 16 in CDCl_3_.

Figure S57. ^1^H-^13^C HSQC NMR (500 MHz) 16 in CDCl_3_.

Figure S58. ^1^H-^13^C HMBC NMR (500 MHz) 16 in CDCl_3_.

### Table S1. Assignment of ^1^H NMR peaks of 16 through combination of ^1^H-^1^H COSY and NOESY.

|  | | | | | | | | | | |  | |
| --- | --- | --- | --- | --- | --- | --- | --- | --- | --- | --- | --- | --- |
| **H_j_** | **H_i2_** | **H_i1_** | **H_h_** | **H_g_** | **H_f_** | **H_e_** | **H_d_** | **H_c_** | **H_b_** | **H_a_** | **δ_ppm_** |  |
| 0.45 | 1.60 | 2.01 | 1.81 | 1.21 | 2.64 | 3.90 | 7.33 | 7.09 | 7.11 | 7.47 |  | **δ_ppm_** |
| x |  | x |  |  |  |  |  |  | v, x |  | 7.47 | **H_a_** |
|  |  |  |  |  |  |  |  |  |  | v, x | 7.11 | **H_b_** |
|  |  |  |  |  |  |  | v, x |  |  |  | 7.09 | **H_c_** |
|  |  |  |  |  |  | x |  | v, x |  |  | 7.33 | **H_d_** |
|  |  |  |  |  |  |  | x |  |  |  | 3.90 | **H_e_** |
|  |  |  |  | v, x |  |  |  |  |  |  | 2.64 | **H_f_** |
|  |  |  |  |  | v, x |  |  |  |  |  | 1.21 | **H_g_** |
|  |  |  |  |  |  |  |  |  |  |  | 1.81 | **H_h_** |
| v, x | v, x |  |  |  |  |  |  |  |  | x | 2.01 | **H_i1_** |
| v, x |  | v, x |  |  |  |  |  |  |  |  | 1.60 | **H_i2_** |
|  | v, x | v, x |  |  |  |  |  |  |  | x | 0.45 | **H_j_** |

v: COSY correlation

x: NOESY correlation

### Table S2. Assignment of ^13^C NMR peaks of 16 through combination of ^1^H-^13^C HSQC and HMBC.

|  | | | | | | | | | | |  | |
| --- | --- | --- | --- | --- | --- | --- | --- | --- | --- | --- | --- | --- |
| **H_j_** | **H_i2_** | **H_i1_** | **H_h_** | **H_g_** | **H_f_** | **H_e_** | **H_d_** | **H_c_** | **H_b_** | **H_a_** | **δ_ppm_** |  |
| 0.45 | 1.60 | 2.01 | 1.81 | 1.21 | 2.64 | 3.90 | 7.33 | 7.09 | 7.11 | 7.47 |  | **δ_ppm_** |
|  |  |  |  |  |  |  |  |  |  | v | 117.5 | **C_a_** |
|  |  |  |  |  |  |  | x |  | v |  | 118.9 | **C_b_** |
|  |  |  |  |  |  |  |  | v |  | x | 118.6 | **C_c_** |
|  |  |  |  |  |  |  | v |  |  |  | 109.4 | **C_d_** |
|  |  |  |  |  |  | v |  |  |  |  | 30.6 | **C_e_** |
|  |  |  |  | x | v |  |  |  |  |  | 19.0 | **C_f_** |
|  |  |  |  | v | x |  |  |  |  |  | 14.7 | **C_g_** |
|  |  |  | v |  |  |  |  |  |  |  | 9.8 | **C_h_** |
| x | v, x | v, x |  |  |  |  |  |  |  |  | 29.3 | **C_i_** |
| v | x | x |  |  |  |  |  |  |  |  | 8.6 | **C_j_** |
|  |  |  |  |  |  |  |  |  |  |  | 123.9 | **C_a'_** |
|  |  |  |  |  |  | x |  |  |  |  | 140.8 | **C_d'_** |
|  |  |  |  |  |  | x |  |  |  |  | 148.1 | **C_e'_** |
|  | x | x |  |  |  |  |  |  |  |  | 122.5 | **C_e''_** |
|  |  |  | x | x | x |  |  |  |  |  | 132.6 | **C_f'_** |
|  |  | x | x |  | x |  |  |  |  |  | 147.5 | **C_h'_** |
| x | x | x | x |  |  |  |  |  |  |  | 54.6 | **C_i'_** |

v: HSQC correlation – (^1^*J*)

x: HMBC correlation – (^1^*J*, ^2^*J*, or ^3^*J*)

# 4. IR spectra

Figure S59. FT-IR of [7](OTf)_2_.

Figure S60. FT-IR of [8](OTf).

# 5. HRMS spectra

Figure S61. HRMS of [1](BF_4_).

Figure S62. HRMS of [2](OTf).

Figure S63. HRMS of [2](BF_4_).

Figure S64. HRMS of [3](BF_4_).

Figure S65. HRMS of [4](OTf).

Figure S66. HRMS of [5](OTf).

Figure S67. HRMS of [6](BF_4_)_2_.

Figure S68. HRMS of [7](OTf)_2_.

Figure S69. HRMS of [8](OTf).

Figure S70. HRMS of 15. Measured (above), simulated (below).

Figure S71. HRMS of 16.

Figure S72. HRMS of 18.

Figure S73. HRMS of 17. Measured (above), calculated (below).

Figure S74. HRMS of 19. Measured (above), calculated (below).

Figure S75. HRMS of 20. Measured (above), calculated (below).

# 6. Crystallography

## 6.1. General

Suitable single crystals of compounds **[2]**(OTf), **[3]**(BF_4_), **[4]**(OTf), **[5]**(OTf), **[6]**(BF_4_)_2_, **[7]**(OTf)_2_, **[8]**(OTf), and **19** were grown from the solvents mentioned in their respective synthetic procedures. Single-crystal X-ray diffraction data collections were performed using Rigaku goniometer diffractometer with graphite mono-chromated Mo Kα radiation (λ = 0.71073 Å). The diffraction intensity details were extracted from the diffraction frames using CrysAlisPro XtalLAB Synergy-S system (version 41.112a) program. The structures were then solved by SHELXT-9740 available in Olex2 crystallographic suite, which located most of the non-hydrogen atoms. Subsequently, least-squares refinements were carried out on F^2^ using SHELXL-Version 2018/3 to locate the remaining non-hydrogen atoms. Non-hydrogen atoms were refined with anisotropic displacement parameters. Hydrogen atoms attached to carbon atoms were fixed in calculated positions. All disorders were handled with SADI, SIMU, EADP, SAME and RIGU constraints and restraints. The crystallographic parameters of each structure are given bellow. All structures are deposited to CCDC and their corresponding deposition numbers are listed in Table S1.

### Table S3. CCDC deposition numbers of XRD structures.

| Compound | CCDC # |
| --- | --- |
| **[2]**(OTf) | 2429275 |
| **[3]**(BF_4_) | 2429276 |
| **[4]**(OTf) | 2429277 |
| **[5]**(OTf) | 2429278 |
| **[6]**(BF_4_)_2_ | 2429280 |
| **[7]**(OTf)_2_ | 2429279 |
| **[8]**(OTf) | 2429281 |
| **19** | 2429282 |

## 6.2. Supplementary XRD structures

Figure S76. XRD structure of complex [8](OTf). H atoms and solvent molecules (benzene) are omitted for clarity.

Figure S77. XRD structure of compound 19. One of two molecules within the asymmetric units is shown.

## 6.3. Crystallographic parameters.

|  | [3](BF_4_) (X = Cl) | [4](OTf) (X = Me) | [5](OTf) (X = H) | [6](BF_4_)_2_ (X = N_MeCN_) |
| --- | --- | --- | --- | --- |
| Rh-S (Å) | 2.1144(9) | 2.1591(8) | 2.182(2) | 2.131(6) |
| Rh-X (Å) | 2.3680(9) | 2.1640(3) | 1.585 | 2.033(2) |
| Rh-P (Å) ^[a]^ | 2.3330(9) | 2.1650(9) | 2.288(3) | 2.348(7) |
| S-Rh-X (°) | 178.70(3) | 178.48(10) | 176.09 | 175.49(7) |
| S-Rh-P (°) ^[a]^ | 87.63(3) | 87.30(3) | 87.71(9) | 85.52(2) |
| P-Rh-P (°) ^[a]^ | 119.83(3) | 119.80(3) | 119.84(10) | 119.39(2) |

Table S4. Selected bond lengths and bond angles Rh(I) sulfonium complexes [3](BF_4_), [4](OTf), [5](OTf), and [6](BF_4_)_2_.

^[a]^ Average value.

### Table S5. Crystallographic parameters of compounds [2](OTf), [3](BF_4_), [4](OTf), and [5](OTf).

|  | **[2]**(OTf) | **[3]**(BF_4_) | **[4]**(OTf) | **[5]**(OTf) |
| --- | --- | --- | --- | --- |
| Identification code | NitsanB7_SP3 | NitsanB53 | nitsanb35 | NitsanB34a SP3RhH |
| Empirical formula | C_57_Cl_4_F_3_H_46_O_3_P_3_S_2_ | C_56_H_44_BCl_7_F_4_P_3_RhS | C_117_H_95_Cl_15_F_6_O_6_P_6_Rh_2_S_4_ | C_67_H_55_F_3_O_3_P_3_RhS_2_ |
| Formula weight | 1134.77 | 1279.75 | 2762.55 | 1225.05 |
| Temperature/K | 149.98(10) | 200.00(10) | 150.15 | 150.00(10) |
| Crystal system | triclinic | monoclinic | monoclinic | monoclinic |
| Space group | P-1 | P2_1_/n | P2_1_/n | P2_1_/n |
| a/Å | 12.7739(2) | 13.5900(3) | 13.8243(3) | 14.220(2) |
| b/Å | 14.3705(2) | 18.9674(4) | 20.0974(3) | 19.1002(17) |
| c/Å | 15.2522(2) | 21.3466(5) | 42.5401(9) | 21.783(4) |
| α/° | 79.8420(10) | 90 | 90 | 90 |
| β/° | 81.4440(10) | 103.039(2) | 98.419(2) | 105.877(17) |
| γ/° | 86.5170(10) | 90 | 90 | 90 |
| Volume/Å^3^ | 2723.54(7) | 5360.6(2) | 11691.7(4) | 5690.7(15) |
| Z | 2 | 4 | 4 | 4 |
| ρ_calc_g/cm^3^ | 1.384 | 1.586 | 1.569 | 1.430 |
| μ/mm^‑1^ | 0.436 | 0.849 | 0.843 | 0.516 |
| F(000) | 1168.0 | 2584.0 | 5592.0 | 2520.0 |
| Crystal size/mm^3^ | 0.18 × 0.16 × 0.1 | 0.146 × 0.09 × 0.046 | 0.12 × 0.07 × 0.07 | 0.132 × 0.045 × 0.041 |
| Radiation | MoKα  (λ = 0.71073) | Mo Kα  (λ = 0.71073) | MoKα  (λ = 0.71073) | Mo Kα  (λ = 0.71073) |
| 2Θ range for data collection/° | 3.928 to 49.998 | 4.002 to 50 | 4.054 to 63.146 | 4.266 to 49.996 |
| Index ranges | -15 ≤ h ≤ 15, -17 ≤ k ≤ 17, -18 ≤ l ≤ 18 | -16 ≤ h ≤ 16, -22 ≤ k ≤ 22, -25 ≤ l ≤ 25 | -18 ≤ h ≤ 17, -19 ≤ k ≤ 28, -44 ≤ l ≤ 56 | -16 ≤ h ≤ 16, -22 ≤ k ≤ 20, -25 ≤ l ≤ 25 |
| Reflections collected | 83749 | 36600 | 93267 | 31982 |
| Independent  reflections | 9581 [R_int_ =0.0346,  R_sigma_ = 0.0169] | 9427 [R_int_ = 0.0466, R_sigma_ = 0.0399] | 33022 [R_int_ = 0.0340, R_sigma_ = 0.0495] | 9930 [R_int_ = 0.1889,  R_sigma_ = 0.1954] |
| Data/restraints/  parameters | 9581/0/622 | 9427/0/658 | 33022/0/1407 | 9930/1/716 |
| Goodness-of-fit  on F^2^ | 1.030 | 1.042 | 1.046 | 1.049 |
| Final R indexes  [I>=2σ (I)] | R_1_ = 0.0551,  wR_2_ = 0.1544 | R_1_ = 0.0455,  wR_2_ = 0.1139 | R_1_ = 0.0496,  wR_2_ = 0.1218 | R_1_ = 0.0972,  wR_2_ = 0.2066 |
| Final R indexes  [all data] | R_1_ = 0.0614,  wR_2_ = 0.1609 | R_1_ = 0.0601,  wR_2_ = 0.1213 | R_1_ = 0.0803,  wR_2_ = 0.1385 | R_1_ = 0.1876,  wR_2_ = 0.2492 |
| Largest  diff. peak/  hole / e Å^-3^ | 1.46/-1.17 | 2.08/-0.73 | 1.37/-0.88 | 1.73/-0.67 |

Table S6. Crystallographic parameters of compounds [6](BF_4_)_2_, [7](OTf)_2_, [8](OTf), and 19.

|  | **[6]**(BF_4_)_2_ | **[7]**(OTf)_2_ | **[8]**(OTf) | **19** |
| --- | --- | --- | --- | --- |
| Identification code | nitsanb35 | NitsanB47 | nitsanb40 | NitsanB41 |
| Empirical formula | C_117_H_95_Cl_15_F_6_O_6_P_6_Rh_2_S_4_ | C_58_H_43_Cl_3_F_6_O_7_P_3_RhS_3_ | C_65_H_51_F_3_O_4_P_4_RhS | C_26_H_24_N_2_ |
| Formula weight | 2762.55 | 1364.27 | 1211.91 | 364.47 |
| Temperature/K | 150.15 | 200.00(10) | 200.15 | 200.00(10) |
| Crystal system | monoclinic | monoclinic | monoclinic | monoclinic |
| Space group | P2_1_/n | P2_1_/n | C2/c | P2_1_/n |
| a/Å | 13.8243(3) | 14.2297(2) | 26.1532(4) | 8.5593(2) |
| b/Å | 20.0974(3) | 16.7190(2) | 16.7657(3) | 31.2972(7) |
| c/Å | 42.5401(9) | 24.9063(3) | 26.9188(5) | 14.6964(4) |
| α/° | 90 | 90 | 90 | 90 |
| β/° | 98.419(2) | 101.8790(10) | 107.751(2) | 94.311(2) |
| γ/° | 90 | 90 | 90 | 90 |
| Volume/Å^3^ | 11691.7(4) | 5798.47(13) | 11241.3(4) | 3925.76(17) |
| Z | 4 | 4 | 8 | 8 |
| ρ_calc_g/cm^3^ | 1.569 | 1.563 | 1.432 | 1.233 |
| μ/mm^‑1^ | 0.843 | 0.695 | 0.514 | 0.072 |
| F(000) | 5592.0 | 2760.0 | 4968.0 | 1552.0 |
| Crystal size/mm^3^ | 0.12 × 0.07 × 0.07 | 0.4 × 0.09 × 0.04 | 0.445 × 0.371 × 0.167 | 0.143 × 0.121 × 0.073 |
| Radiation | MoKα  (λ = 0.71073) | Mo Kα  (λ = 0.71073) | MoKα  (λ = 0.71073) | Mo Kα  (λ = 0.71073) |
| 2Θ range for data collection/° | 4.054 to 63.146 | 4.136 to 64.77 | 4.674 to 50 | 4.792 to 64.874 |
| Index ranges | -18 ≤ h ≤ 17, -19 ≤ k ≤ 28, -44 ≤ l ≤ 56 | -20 ≤ h ≤ 19, -24 ≤ k ≤ 24, -37 ≤ l ≤ 36 | -31 ≤ h ≤ 30, -19 ≤ k ≤ 19, -32 ≤ l ≤ 31 | -12 ≤ h ≤ 12, -47 ≤ k ≤ 45, -21 ≤ l ≤ 21 |
| Reflections collected | 93267 | 130823 | 50162 | 71360 |
| Independent  reflections | 33022 [R_int_ = 0.0340, R_sigma_ = 0.0495] | 18256 [R_int_ = 0.0369, R_sigma_ = 0.0263] | 9887 [R_int_=0.0317,  R_sigma_ = 0.0220] | 12381 [R_int_ = 0.0461, R_sigma_ = 0.0394] |
| Data/restraints/  parameters | 33022/0/1407 | 18256/0/758 | 9887/0/692 | 12381/0/511 |
| Goodness-of-fit  on F^2^ | 1.046 | 1.045 | 1.031 | 1.043 |
| Final R indexes  [I>=2σ (I)] | R_1_ = 0.0496,  wR_2_ = 0.1218 | R_1_ = 0.0315,  wR_2_ = 0.0764 | R_1_ = 0.0285,  wR_2_ = 0.0718 | R_1_ = 0.0504,  wR_2_ = 0.1176 |
| Final R indexes  [all data] | R_1_ = 0.0803,  wR_2_ = 0.1385 | R_1_ = 0.0457,  wR_2_ = 0.0837 | R_1_ = 0.0331,  wR_2_ = 0.0755 | R_1_ = 0.0804,  wR_2_ = 0.1289 |
| Largest  diff. peak/  hole / e Å^-3^ | 1.37/-0.88 | 0.80/-0.55 | 0.50/-0.43 | 0.29/-0.23 |

# 7. Computational data

## 7.1. Input templates

The following input templates were used for the calculations

a) *Geometry optimization*

#P wb97xD/def2tzvp opt=(cartesian,calcfc,MaxCycle=500) Freq Int=Ultrafine

\# SCF=(xqc, Fermi) SCFCyc=180 Nosymm

b) *NBO 7 calculations*

#P wb97xD/def2tzvp Int=Ultrafine pop=(NBO7Read,savenlmos)

…

$NBO NLMO NBOSUM BNDIDX $END

## 7.2. Summary of the computational results

Figure S78. Model structures of isostructural series of Rh(I) complexes of coordinated acetone molecule with Si-, P-, and S-based tripodal ligands used for computational studies.

|  | [(EP_3_)Rh(CO)]^n+^ (n = 0, 1 or 2) | | | | | |
| --- | --- | --- | --- | --- | --- | --- |
|  | E=Si^-^ (**10***) ^[b]^ | | E=P (**8***) | | E=S^+^ (**7***) | |
|  | Optimized | XRD | Optimized | XRD | Optimized | XRD |
| Rh-E (Å) | 2.379 | 2.3308(6) | 2.284 | 2.3605(5) | 2.257 | 2.2047(4) |
| Rh-P_eq_ Å) ^[a]^ | 2.295 | 2.312(6) | 2.315 | 2.344 (5) | 2.335 | 2.360 (4) |
| Rh-C (Å) | 1.930 | 1.921(3) | 1.916 | 1.902(2) | 1.892 | 1.8833(4) |
| C-O (Å) | 1.140 | 1.137(3) | 1.132 | 1.122(3) | 1.127 | 1.125(2) |
| E-Rh-C (°) | 179.97 | 178.11(9) | 179.98 | 175.92 (8) | 179.99 | 177.30(6) |
| E-Rh-P_eq_ (°) ^[a]^ | 81.82 | 81.96(9) | 83.65 | 83.76 (18) | 85.07 | 85.28 (14) |
| P_eq_-Rh-P_eq_ (°) ^[a]^ | 117.77 | 118.06(9) | 118.60 | 118.83 (19) | 119.12 | 119.31(14) |

Table S7. Selected bond lengths and angles in optimized model structures 10*, 8*, and 7* compared to those in the XRD structures of complexes 10, [8](OTf), and [7](OTf)_2_.

^[a]^ Average value. ^[b]^ The data is taken from ref. [46]

|  | [(EP_3_)Rh(Acetone)]^n+^ (n = 0, 1 or 2) | | |
| --- | --- | --- | --- |
|  | E=Si^-^ (**21***) | E=P (**22***) | E=S^+^ (**23***) |
| Rh-E (Å) | 2.264 | 2.188 | 2.160 |
| Rh-P_eq_ Å) ^[a]^ | 2.295 | 2.325 | 2.357 |
| Rh-O (Å) | 2.331 | 2.191 | 2.099 |
| C=O (Å) | 1.214 | 1.221 | 1.231 |
| E-Rh-O (°) | 176.00 | 175.12 | 174.33 |
| E-Rh-P_eq_ (°) ^[a]^ | 83.31 | 84.07 | 84.92 |
| P_eq_-Rh-P_eq_ (°) ^[a]^ | 118.66 | 118.95 | 119.22 |

### Table S8. Selected bond lengths and angles in the optimized model complexes 21*, 22* and 23*.

^[a]^ Average value.

|  | [(EP_3_)Rh(CO)]^n+^ (n = 0, 1 or 2) | | | [(EP_3_)Rh(Acetone)]^n+^ (n = 0, 1 or 2) | | |
| --- | --- | --- | --- | --- | --- | --- |
|  | E=Si^-^ (**10***) | E=P (**8***) | E=S^+^ (**7***) | E=Si^-^ (**21***) | E=P (**22***) | E=S^+^ (**23***) |
| Rh | -0.435 | -0.349 | -0.271 | -0.388 | -0.206 | -0.050 |
| E | 1.286 | 1.144 | 0.990 | 1.409 | 1.257 | 1.079 |
| P_eq_ ^[a]^ | 0.457 | 0.447 | 0.430 | 0.428 | 0.417 | 0.398 |
| C_CO_ | 0.479 | 0.561 | 0.624 | - | - | - |
| O_CO_ | -0.493 | -0.433 | -0.384 | - | - | - |
| C_Acetone_ | - | - | - | 0.612 | 0.639 | 0.661 |
| O_Acetone_ | - | - | - | -0.562 | -0.565 | -0.557 |

### **Table S9**. Natural Population charges of selected atoms in Rh(CO) and Rh(Acetone) complexes.

^[a]^ Average value.

Table S10. Selected bond Wiberg indices^[85, 86]^ in Rh(CO) and Rh(Acetone) complexes.

|  | [(EP_3_)Rh(CO)]^n+^ (n = 0, 1 or 2) | | | [(EP_3_)Rh(Acetone)]^n+^ (n = 0, 1 or 2) | | |
| --- | --- | --- | --- | --- | --- | --- |
|  | E=Si^-^ (**10***) | E=P (**8***) | E=S^+^ (**7***) | E= Si^-^ (**21***) | E=P (**22***) | E=S^+^ (**23***) |
| E-Rh | 0.458 | 0.479 | 0.394 | 0.683 | 0.727 | 0.588 |
| Rh-P_eq_ ^[a]^ | 0.343 | 0.347 | 0.350 | 0.359 | 0.354 | 0.358 |
| Rh-C_CO_ | 0.637 | 0.683 | 0.783 | - | - | - |
| C≡O | 2.083 | 2.167 | 2.211 | - | - | - |
| Rh-O_Acetone_ | - | - | - | 0.126 | 0.207 | 0.327 |
| C=O | - | - | - | 1.753 | 1.689 | 1.611 |

^[a]^ Average value.

### Table S11. NLMOs representing the major E-Rh bonding interaction in the optimized model complexes 10*, 8* and 7* (isovalue = 0.03).

|  | [(EP_3_)Rh(CO)]^n+^ (n = 0, 1 or 2) | | |
| --- | --- | --- | --- |
|  | E=Si^-^ (**10***) | E=P (**8***) | E=S^+^ (**7***) |
|  |  |  |  |
| E (%) | 30.78 | 51.91 | 70.35 |
| Rh (%) | 63.63 | 44.68 | 27.59 |

### Table S12. NLMOs representing the major E-Rh bonding interaction in the optimized model complexes 21*, 22* and 23* (isovalue = 0.03).

|  | [(EP_3_)Rh(Acetone)]^n+^ (n = 0, 1 or 2) | | |
| --- | --- | --- | --- |
|  | E= Si^-^ (**21***) | E=P (**22***) | E=S^+^ (**23***) |
|  |  |  |  |
| E (%) | 32.70 | 53.28 | 70.69 |
| Rh (%) | 63.18 | 43.78 | 27.44 |

# 8. Additional References

[74]	Arnáiz, F. J. The Preparation of TlBF_4_. *J. Chem. Educ.*, **1997**, *74* (11), 1332.

[75]	Kulzick, M. A.; Price, R. T.; Andersen, R. A.; Muetterties, E. L. Bridging Alkyls in *d*-Transition Metal Chemistry: Reaction of (COD)_2_RH_2_(μ-R)_2_ with Lewis Bases to Give (COD)Rh(R)(L) and Their Reaction with Aromatic Hydrocarbons. *J. Organomet. Chem.*, **1987**, *333* (1), 105–118.

[76]	Annibale, V. T.; Song, D. Reaction of Dinuclear Rhodium 4,5-Diazafluorenyl-9-Carboxylate Complexes with H_2_ and CO_2_. *Organometallics*, **2014**, *33* (11), 2776–2783.

[77]	Harris, R. K.; Becker, E. D.; Menezes, S. M. C. de; Granger, P.; Hoffman, R. E.; Zilm, K. W. Further Conventions for NMR Shielding and Chemical Shifts (IUPAC Recommendations 2008). *Pure Appl. Chem.*, **2008**, *80* (1), 59–84.

[78]	Frisch, M. J.; Trucks, G. W.; Schlegel, H. B.; Scuseria, G. E.; Robb, M. A.; Cheeseman, J. R.; Scalmani, G.; Barone, V.; Petersson, G. A.; Nakatsuji, H.; et al. Gaussian 16 Rev. C.01, **2016**.

[79]	Chai, J.-D.; Head-Gordon, M. Long-Range Corrected Hybrid Density Functionals with Damped Atom–Atom Dispersion Corrections. *Phys Chem Chem Phys*, **2008**, *10* (44), 6615–6620.

[80]	Weigend, F.; Ahlrichs, R. Balanced Basis Sets of Split Valence, Triple Zeta Valence and Quadruple Zeta Valence Quality for H to Rn: Design and Assessment of Accuracy. *Phys. Chem. Chem. Phys.*, **2005**, *7* (18), 3297–3305.

[81]	E. D. Glendening, J, K. Badenhoop, A. E. Reed, J. E. Carpenter, J. A. Bohmann, C. M. Morales, P. Karafiloglou, C. R. Landis, and F. Weinhold. NBO 7.0, **2018**.

[82]	Lee, S. O.; Choi, J.; Kook, S.; Lee, S. Y. Lewis Acid-Catalyzed Double Addition of Indoles to Ketones: Synthesis of Bis(Indolyl)Methanes with All-Carbon Quaternary Centers. *Org. Biomol. Chem.*, **2020**, *18* (44), 9060–9064.

[83]	Soni, S.; Teli, S.; Teli, P.; Agarwal, S. Revolutionizing Green Catalysis: A Novel Amla Seed Derived Biochar Modified g-C_3_N_4_·SO_3_H Catalyst for Sustainable and Versatile Synthesis of Bis-Indoles. *Nanoscale Adv.*, **2025**, *7* (6), 1603–1616.

[84]	Jaiswal, K.; Roy, K.; De, M. Effect of Metal Doping on the Catalytic Activity of MoS_2_: Access to High-Spin Co (III) Species In Situ toward the Synthesis of Bisindolylmethanes and a Study of Their Bioactivity. *ACS Appl. Mater. Interfaces*, **2025**, *17* (24), 35585–35596.

[85]	Wiberg, K. B. Application of the Pople-Santry-Segal CNDO Method to the Cyclopropylcarbinyl and Cyclobutyl Cation and to Bicyclobutane. *Tetrahedron*, **1968**, *24* (3), 1083–1096.

[86]	Harper, L. K.; Shoaf, A. L.; Bayse, C. A. Predicting Trigger Bonds in Explosive Materials through Wiberg Bond Index Analysis. *ChemPhysChem*, **2015**, *16* (18), 3886–3892.
